# ODFM, an omics data resource from microorganisms associated with fermented foods

**DOI:** 10.1038/s41597-021-00895-x

**Published:** 2021-04-20

**Authors:** Tae Woong Whon, Seung Woo Ahn, Sungjin Yang, Joon Yong Kim, Yeon Bee Kim, Yujin Kim, Ji-Man Hong, Hojin Jung, Yoon-E Choi, Se Hee Lee, Seong Woon Roh

**Affiliations:** 1 Microbiology and Functionality Research Group, World Institute of Kimchi, Gwangju, 61755 Korea; 2 Insilicogen Inc., Gyeonggi-do, 16954 Korea; 3 Division of Environmental Science and Ecological Engineering, Korea University, Seoul, 02841 Korea

**Keywords:** Bacterial genomics, Archaeal genomics

## Abstract

ODFM is a data management system that integrates comprehensive omics information for microorganisms associated with various fermented foods, additive ingredients, and seasonings (e.g. kimchi, Korean fermented vegetables, fermented seafood, solar salt, soybean paste, vinegar, beer, cheese, sake, and yogurt). The ODFM archives genome, metagenome, metataxonome, and (meta)transcriptome sequences of fermented food-associated bacteria, archaea, eukaryotic microorganisms, and viruses; 131 bacterial, 38 archaeal, and 28 eukaryotic genomes are now available to users. The ODFM provides both the Basic Local Alignment Search Tool search-based local alignment function as well as average nucleotide identity-based genetic relatedness measurement, enabling gene diversity and taxonomic analyses of an input query against the database. Genome sequences and annotation results of microorganisms are directly downloadable, and the microbial strains registered in the archive library will be available from our culture collection of fermented food-associated microorganisms. The ODFM is a comprehensive database that covers the genomes of an entire microbiome within a specific food ecosystem, providing basic information to evaluate microbial isolates as candidate fermentation starters for fermented food production.

## Introduction

Advances in next-generation sequencing technology have led to the rapid expansion of microbial genome sequence data. Easy access, as well as convenient analytical tools, have enabled the exploration of microbial communities in various environmental samples. However, efficient resource usage is becoming increasingly difficult because of the rapid accumulation of sequencing data. Environmental microbiomes in fermented foods, the mammalian gut, and soils comprise not only bacteria, but also archaea, eukaryotic microorganisms, and viruses^1–3^. These microbial entities are all essential in determining the microbial signature and thus, the inherent characteristics of a given ecosystem. In this context, a comprehensive database covering all genomes of a microbiome within a specific ecosystem would aid in improving our understanding of the complex interactions among the microbial populations.

Fermented foods are an integral part of the global human diet. Microbial entities in fermented foods include bacteria, archaea, yeasts, and viruses. Microbial activities, as well as the type of raw materials, ultimately determine the nutritional and organoleptic properties, quality, and safety of the fermentation product^4,5^. Given that consumers and manufacturers alike are interested in tasty, high-quality foods^6^ as well as the reliability of geographic origins (i.e. no false indication of the origin of the product)^1^, providing standardised microbial profiles and/or genome information for key microorganisms during the fermentation process is important for ensuring the high quality of fermentation products.

Kimchi is a traditional Korean food prepared by fermentation of vegetables, such as kimchi cabbage, along with various added ingredients and seasonings. The global annual consumption of kimchi is 1,500,000 tons^1^. Like other fermented foods, kimchi shows the presence of a distinct microbial community^4,5^. Taxonomic studies using culture-dependent and -independent (e.g. bacterial 16 S rRNA gene sequencing) approaches have revealed that lactic acid bacteria (LAB), including *Leuconostoc*, *Lactobacillus*, and *Weissella*, are mainly responsible for kimchi fermentation^7–9^.

We have developed the Omics Database of Fermentative Microbes (ODFM), a data management system that integrates comprehensive omics information for fermentative microorganisms at the World Institute of Kimchi funded by the Korean government. The ODFM offers not only curated omics sequences of fermented food-associated bacteria, archaea, eukaryotic microorganisms, and viruses, but also several analytical tools that enable gene diversity and taxonomic analyses of an input query on the database at the whole genome level. Our knowledgebase is valuable to researchers who are interested in the functions and spatiotemporal dynamics of microbiomes in fermented foods. In particular, it provides basic information to evaluate microbial strains isolated from fermented foods as candidate starters in terms of food safety and sanitation.

## Results

### System design and data registration

The ODFM is a web-based application developed in compliance with the HyperText Markup Language (HTML) 5 web standards and, thus, is supported by most web browsers. The program was designed based on the Representational State Transfer (REST) service architecture to support use on various devices, including desktop computers and mobile devices. To support stable web service in a cloud-based service environment, the ODFM is hosted on four servers (web, web application, database, and storage servers). Key specifications for each server are summarised in Table 1.

**Table 1:** Features of the ODFM.

Category	Features
**System environment**	
Operating system	Centos (v6.5)
Java runtime environment	Java Development Kit (v1.8)
Web application	Apache (v2.2.15) and Tomcat server (v7.0)
Database operation	MySQL (v5.7)
**Database content**	
Genome	
Bacteria	62 complete and 69 draft genomes covering 96 (sub)species
Archaea	7 complete and 31 draft genomes covering 36 species
Unicellular eukaryotes	14 complete and 14 draft genomes covering 9 species
Metagenome	10 total and 60 viral metagenomes (kimchi)
Metataxonome	113 bacterial metataxonomes (kimchi, fermented seafood, and soybean paste)
(Meta)transcriptome	5 metatranscriptome (kimchi) and 4 archaeal transcriptome
Metabolome	7 metabolomes (kimchi and fermented seafood)
**Functionality**	
Browsing	JBrowse and GView Java package
Tools	Sequence similarity search (BLAST) and genomic relatedness analysis (ANI)

**Fig. 1:**
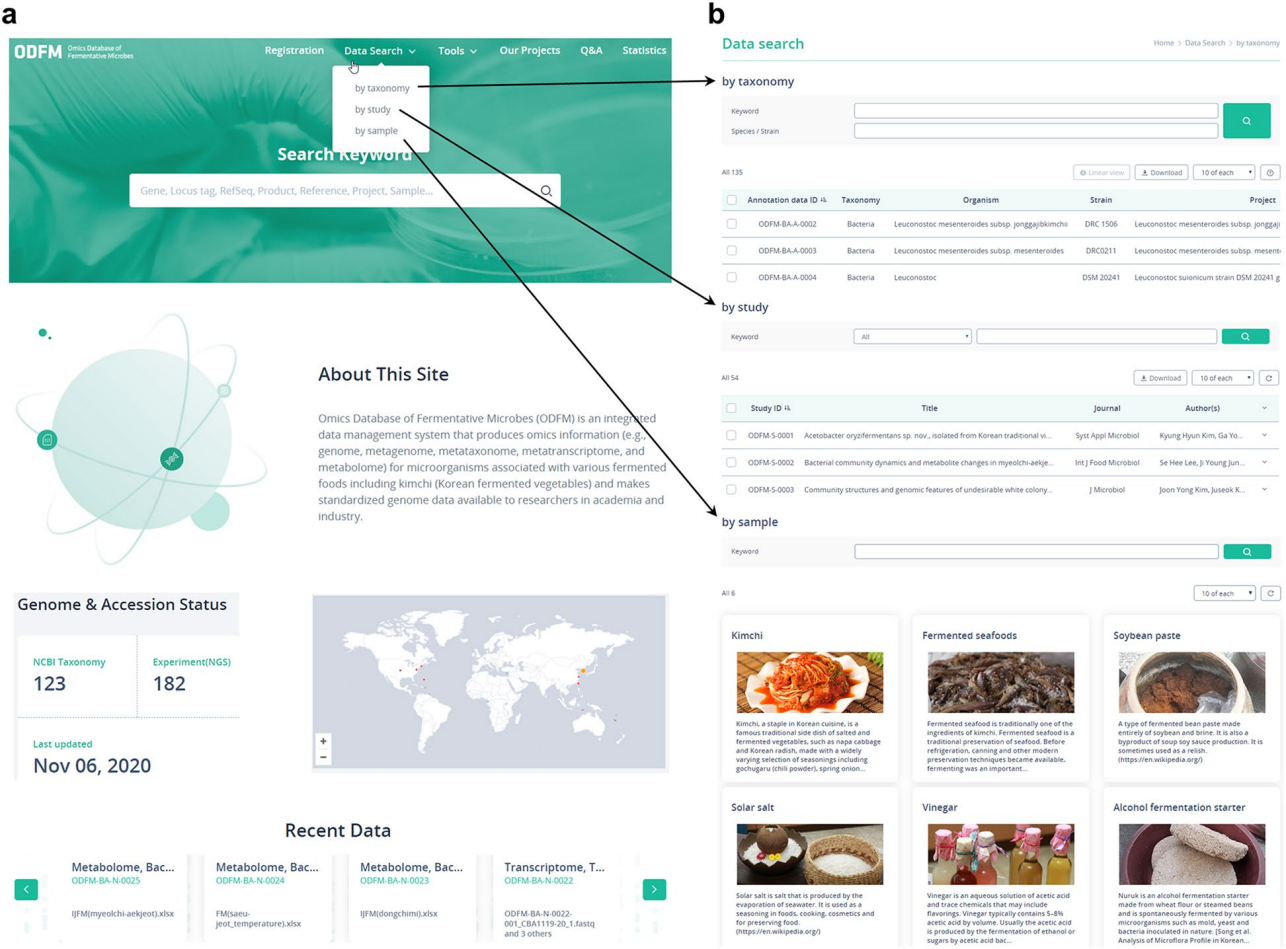
Search functions in the ODFM knowledgebase. (**a)** Front page of the ODFM knowledgebase. (**b)** Basic search functions of the ODFM. By selecting one of three search functions on the data search tab, users can search omics data categorised by taxonomy, study, and sample.

The software architecture of the ODFM consists of client, server, and database modules. The client module uses Google’s AngularJS (version 1.7) as a front-end framework to support cross browsing. The server module operates on JAVA (https://www.oracle.com/technetwork/java/index.html)-implemented Spring framework (https://spring.io) and is additionally equipped with the open-source programs Python (https://www.python.org/) and FastQC (https://www.bioinformatics.babraham.ac.uk/projects/fastqc/). The database module uses MySQL (https://www.mysql.com) to manage the database, while the ODFM user interface architecture consists of Registration, Data Search, Tools, Our Projects, Statistics, and Q&A (Fig. 1a).

To improve data search and the accuracy of search results, submitted genome data and metadata are stored on the storage server and the database server, respectively. The system administrator processes the verification, approval, and release of the registered data. In the data verification step, validation of the file format and conversion of the registered data files are processed in batch using a back-end module. Once the processing is completed, the registered data are presented to the user in the web browser. The ODFM provides an interface to report data, including sequence files, annotated files, and results of sequence quality control according to the data file types. In addition, JBrowse and GView are integrated into the system to provide microbial genome analysis services via the genome browser. A diagram describing the data registration is provided in Supplementary Fig. S1.

### Database content

The ODFM database currently contains 131 complete/draft bacterial genome sequences covering 38 genera with 96 (sub)species, of which 24 (sub)species belong to the genus *Lactobacillus*^10^, 12 to the genus *Leuconostoc*^11–16^, seven to the genus *Acetobacter*^17^, six to the genus *Staphylococcus*^18^, five to the genus *Enterococcus*, four (sub)species to each of the genera *Pediococcus* and *Weissella*^19,20^, three to the genus *Lactococcus*, two (sub)species to each of the genera *Brachybacterium*, *Clostridium*, *Corynebacterium*, and *Pseudomonas*, and one species to each of the genera *Alishewanella*^21^, *Bacillus*^22,23^, *Brevibacterium*, *Dietzia*, *Escherichia*, *Glutamicibacter*, *Hafnia*, *Halomonas*, *Lentibacillus*, *Listeria*, *Megasphaera*, *Microbacterium*, *Morganella*, *Mycetocola*, *Oceanobacillus*, *Paracoccus*, *Pectinatus*, *Pistricoccus*, *Propionibacterium*, *Salimicrobium*^24^, *Streptococcus*, *Tetragenococcus*, and *Vibrio*. As for archaea, 38 complete/draft genome sequences of extremely halophilic archaea (19 genera with 36 species) are available, including seven species belonging to the genus *Haloarcula*^25^, four belonging to the genus *Halorubrum*^26^, three belonging to each of the genera *Haloferax* and *Natronomonas*, two belonging to each of the genera *Halapricum*^27^, *Halobacterium*^28^, *Halolamina*^29^ and *Haloplanus*, and one species belonging to each of the genera *Haladaptatus*^30^, *Halalkalicoccus*, *Halarchaeum*, *Halobellus*^31^, *Halococcus*, *Halogeometricum*, *Halopenitus*, *Halorhabdus*, *Halostella*, *Haloterrigena*^32^, and *Natrinema*^33^. As for eukaryotic microorganisms, 28 genome sequences are currently available, including five undesirable white colony-forming yeasts of the species *Candida*, *Hanseniaspora*, *Kazachstania*, *Pichia*, and *Yarrowia*. These spoilage yeasts can grow on the surface of the kimchi and affect its odour, appearance, and texture^34^. Genome sequences of *Brettanomyces*-, *Penicillium*-, and *Saccharomyces*-belonging species isolated from beer, cheese, and sake, respectively, are available. The database also contains 70 metagenomes, 113 bacterial metataxonomes, nine (meta)transcriptomes, and seven metabolomes for various fermented foods (Table 1). The viral metagenomic sequences have been deposited in the European Bioinformatics Institute (EMBL-EBI) database^35^ and are available under accession number PRJEB23957. Details on ODFM database contents are provided in Supplementary Table S1^1,7,9–14,16–34,36–108^.

### Functional omics archive for fermented food-associated microorganisms

The primary purpose of the ODFM is to provide integrative functional omics information on fermented food-associated microorganisms. The ODFM and online resource provide omics information for microbial isolates from food materials (e.g. kimchi, fermented seafood, solar salt, soybean paste, vinegar, beer, cheese, sake, and yogurt). Recent microbial community analyses based on metataxonomics have revealed that hundreds of bacterial operational taxonomic units/amplicon sequence variants can be detected in fermented foods, and that the number of species varies according to the fermentation process^1,7^. To cover the entire microbial populations involved in food fermentation, since 2018, we have been constantly isolating and sequencing fermented food-associated microorganisms, and updating the database with new data to expand the ODFM archive.

#### Search function

For easy access of omics information, several search tools with simple (i.e. exact-match keyword) and lexical (i.e. partial-match keyword) search options are available on the front page (Fig. 1a). These tools allow users to search for different combinations of search terms. Users can search microbial taxa at the species and strain levels. Once a taxon name is provided by users, the system returns categorised search results (Supplementary Fig. S2a). Results are presented in a tabular format, with each row depicting a microbial taxon that contains the query gene. In the annotation data detail page, a split function in each row shows detailed information on the submitters, isolation sources, sequencing, and annotation results (Supplementary Fig. S2b). The annotation results presented in columns link to both available datasets and additional functions, such as genome viewer. For easy integration of omics data, the ODFM provides three basic search functions. Users can search omics data by taxonomy (i.e. bacteria, archaea, eukaryotic microorganisms, and viruses), study (i.e. publicly available studies highlighting the fermentative microbes), and sample (e.g. kimchi, fermented seafood, solar salt, soybean paste, vinegar, beer, cheese, sake, and yogurt) (Fig. 1b).

#### Genome browsing function

**Fig. 2:**
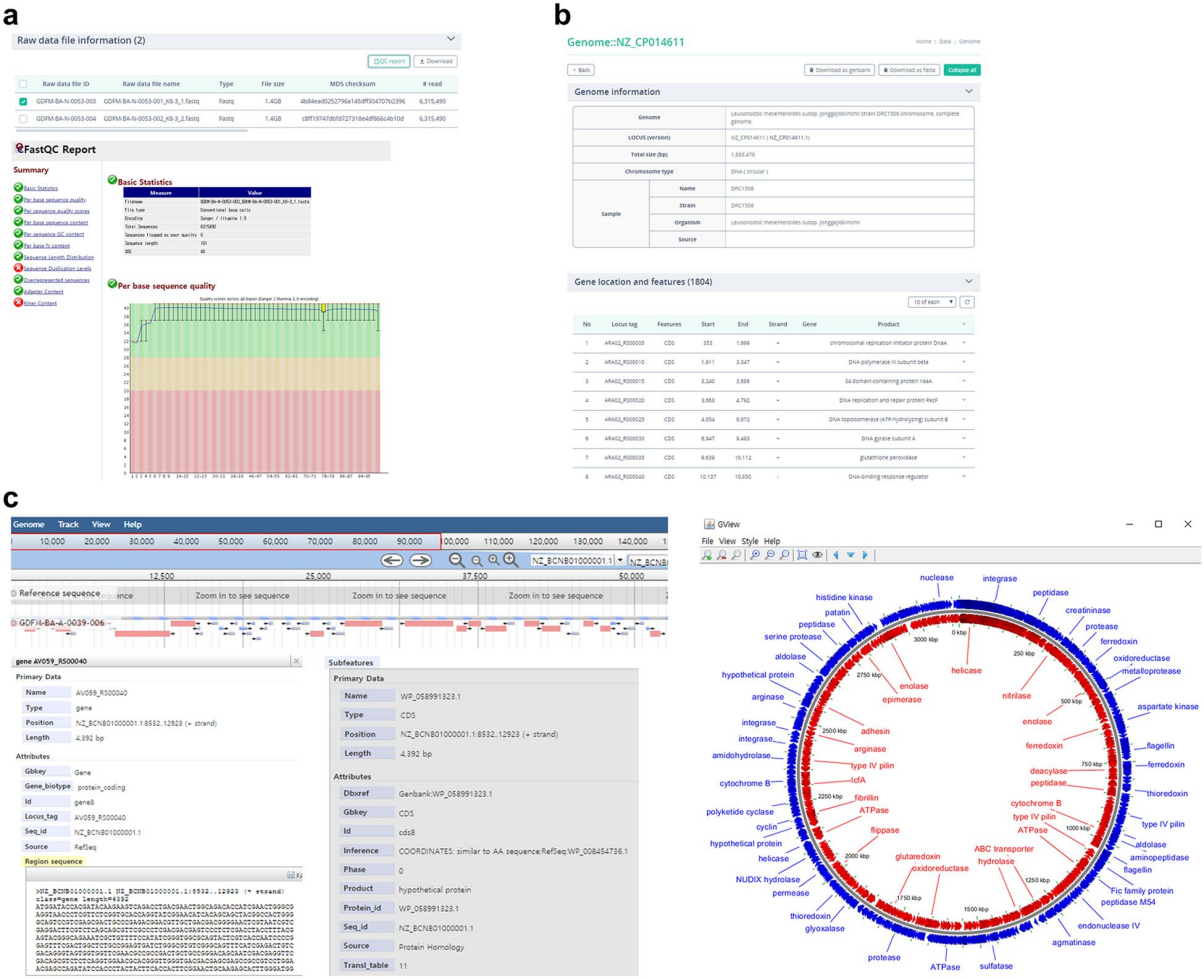
Genome browsing function in the ODFM. (**a**) Screen image of raw data details comprising raw data information and QC information tabs. The raw data file information tab provides basic information on raw data. The QC report tab utilises FastQC to facilitate simple quality control checks of raw sequence data. (**b**) Details on annotation results, including location and product name of the gene/CDS in table format by using the GenBank (gbk) file format. (**c**) The ODFM converts the GFF file to graphical genome data with linear (left) and circular genome views (right).

In the data search page, we provide several browsing functions for annotation data of the database resources. The raw data file information tab provides basic information on raw data (i.e. information regarding the experiment, library preparation, sequencing, and FASTQ file). The QC report tab utilises FastQC (https://www.bioinformatics.babraham.ac.uk/projects/fastqc/) to facilitate simple quality control checks of raw sequence data (Fig. 2a). On the sequence information tab, the browser provides basic (i.e. FASTA) as well as detailed information about annotation data (Fig. 2b). The program uses the GenBank (gbk) file format and returns annotation results, such as location and product name of the gene/coding sequence (CDS). Furthermore, the ODFM provides linear and circular genome views representing graphical genome data based on GFF file information (Fig. 2c). The linear view utilises JBrowse^109^, enabling users to browse local annotation results, while the circular view provides microbial genome visualisation in a circular context with an interactive pan and zoom interface using the GView JAVA package^110^.

#### Archive expansion

We are currently seeking ways to expand the ODFM archive. Though the system currently does not allow data registration by other researchers, we are willing to accommodate deposition of fermented food-associated omics data generated by others upon request. In addition to the genome resources, the ODFM currently covers various outputs of genome annotation results generated from other databases, such as Kyoto Encyclopaedia of Genes and Genomes (KEGG)^111^ and Clusters of Orthologous Groups of proteins (COGs)^112^. In our projects tab on the front page, a list of fermented food-associated microbial studies is provided (Supplementary Fig. S3).

### Comparative genomics

The microbial resources included in the ODFM are all candidate culture starters for fermented foods. Given that metabolic capabilities (e.g. lactate, lactose, and citrate metabolism) and resistance to bacteriophages, but not antibiotic resistance and virulence potential, are desirable functions for a candidate starter^113,114^, preliminary screening by means of comparative genomics analysis between query and subject genome resources can be a practical way to select strains for the production of fermented foods. Accordingly, several open-source analytical tools for comparative genomics analysis are integrated in the ODFM.

#### Sequence similarity search against the ODFM

**Fig. 3:**
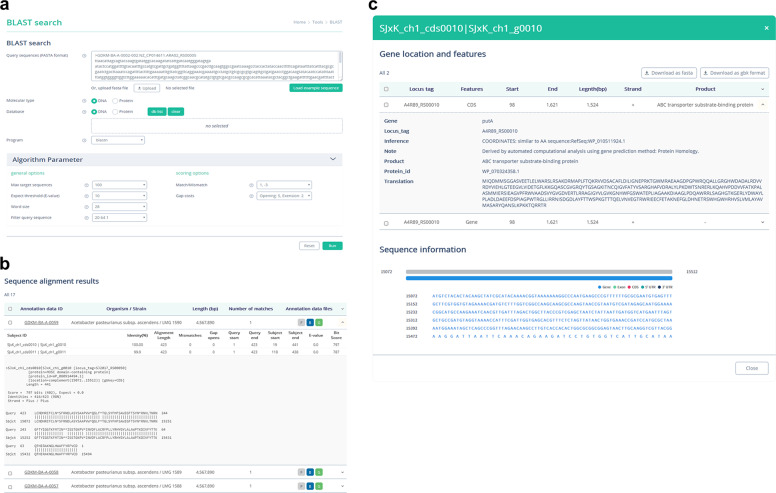
BLAST search function in the ODFM (**a**) Screen image of the BLAST search tab. Users can provide nucleotide/amino acid sequences, and select a BLAST program (blastn, blastp, blastx, tblastnl, or tblastx), expected threshold, and filter query sequence (true or false). (**b,c**) The system returns alignment results in order of match (**b**), and provides sequence/CDS information for the annotated data as a downloadable text format (**c**).

A sequence similarity search against public nucleotide databases is generally the first step in the identification of microbial isolates. The ODFM provides the Basic Local Alignment Search Tool (BLAST)^115^ search-based local alignment function. Once nucleotide/amino acid sequences are provided by users, the system aligns the query sequences with the local DNA/protein database (Fig. 3a). The system returns alignment results with statistical indicators, including bit score and E-value (Fig. 3b), and provides sequence/CDS information for the annotated data in a downloadable text format (Fig. 3c). Given that the ODFM comprises primarily fermentation-associated microbial genomes, this function is particularly helpful for an initial similarity search of strong candidate fermentative starter strains.

#### Genetic relatedness analysis

**Fig. 4:**
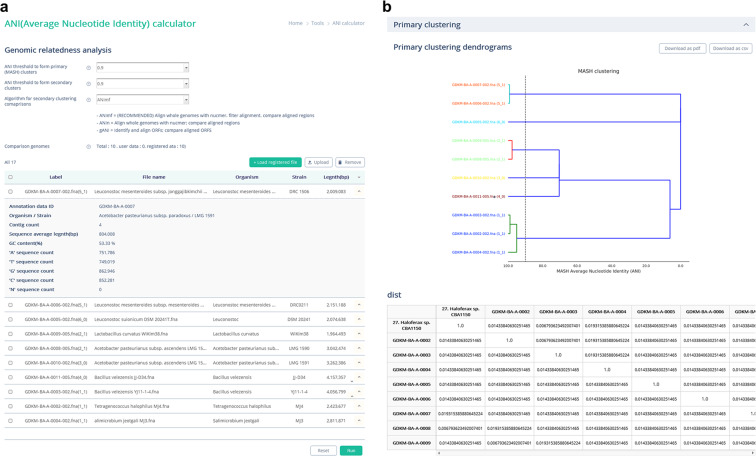
ANI calculator function in the ODFM. (**a**) Screen image of the ANI calculator tab. Users can form comparative datasets by selecting FASTA files from the registered data in the ODFM or by uploading their own sequences. (**b**) The calculation results are returned as a downloadable table and image.

For average nucleotide identity (ANI)-based genome clustering and genetic relatedness measurement, the dRep tool^116^ was integrated into the ODFM. The ANI value is calculated from two genome sequences. Users can create comparative datasets by selecting FASTA files from the registered data in the ODFM or by uploading their own sequences (Fig. 4a). The query genome sequence (complete or draft) is cut into 1,020 bp-long sequences^117^, and each fragment is annotated against the whole sequence of the subject genome. Using the MinHash distance to estimate similarity between genomes^118^, the dRep tool calculates nucleotide identity between each of the query fragments and the subject genome and returns the ANI value, allowing for simple and standardised procedures for genome-related analysis of microbial isolates with closely related strains. The results are provided as a downloadable table and image (Fig. 4b).

## Discussion

We developed the ODFM, a web-based knowledgebase featuring archival and analytical functions for genome data for bacteria, archaea, eukaryotic microorganisms, and viruses associated with fermented foods. The ODFM is freely available on the website https://odfm.wikim.re.kr. This easily accessible online-browsable resource facilitates rapid and functional explorations of genomes of fermentation-associated microorganisms. Genome sequence and annotation results for reference microorganisms, as well as analytical results, are directly downloadable. All microbial strains registered in the ODFM will be made available. Our team operates a culture collection of fermented food-associated microorganisms, the Microorganism and Gene Bank (https://mgb.wikim.re.kr), at the World Institute of Kimchi.

By using the omics resources combined with the search tools, users are able to evaluate microbial strains isolated from fermented foods as candidate starters, and/or select microbial strain(s) among the deposited resources for use as starters. However, the process of fermentation is difficult to control because the fermentation phenotypes of different isolates are influenced by environmental conditions (e.g., temperature, humidity, and type of ingredients) and interactions with other, pre-colonised microbial communities. Previous studies have reported findings that allow linking of certain fermentative microbes with expected key features/metabolites in kimchi fermentation. *Leuconostoc* and *Lactobacillus* species are the major mannitol- and gamma-aminobutyric acid-producing LAB, respectively^119^. *Lactococcus* and some *Lactobacillus* species are homo-fermentative LAB responsible for the production of lactate from pyruvate by lactate dehydrogenase^120^. *Leuconostoc mesenteroides*, *Lactobacillus sakei*, and *Weissella koreensis* convert pyruvate to diacetyl/acetoin by using acetolactate synthase, acetolactate decarboxylase, and diacetyl reductase and thus contribute to the flavour of kimchi^15,20,121^.

We expect the ODFM to provide a framework for the analysis of genome characteristics of microorganisms isolated from various fermented foods. To increase the usage of the data and information contained in the ODFM knowledgebase, we will continuously improve the features and performance of each function. By adding categories of fermented foods based on global consumer preferences and encompassing microbial resources, our long-term goal with the ODFM is to facilitate the genomic characterisation of food microorganisms and their application as fermentation starters, as well as further functional probiotics and biological agents.

## Supplementary information

Supplementary Information

## Data Availability

The ODFM is licensed under a Creative Commons Attribution 4.0 International License. The genome sequences in the ODFM are freely available on the website https://odfm.wikim.re.kr. All genome sequences have been deposited in NCBI GenBank and are available under the accession numbers listed in Supplementary Table S1. The viral metagenomic sequences have been deposited in the European Bioinformatics Institute (EMBL-EBI) database^35^ and are available under accession number PRJEB23957. We plan to deposit additional genome sequences for fermentative microbes to a member of the INSDC (http://www.insdc.org/) to promote sharing activities in the genomics community.

## Code availability

The code used to build the systemic architecture of the GDKM is available on GitHub: https://github.com/yang4851/gdkm.
