# Supplementary material for: ODFM, an omics data resource from microorganisms associated with fermented foods
Source: Sci Data. 2021 Apr 20;8:113. doi: 10.1038/s41597-021-00895-x (PMC8058077; doi:10.1038/s41597-021-00895-x)
Supplement: Supplementary file 1 — Supplementary Information [file 41597_2021_895_MOESM1_ESM.docx]

**The Supplementary Information file includes:**

Supplementary Figures S1 to S3

Supplementary Table S1

**
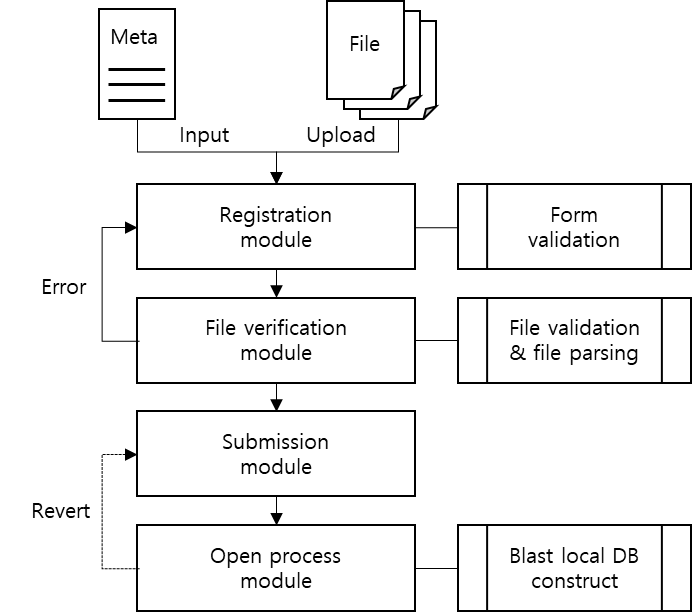
**

**Supplementary Fig. S1. Diagram of data registration in the ODFM.**


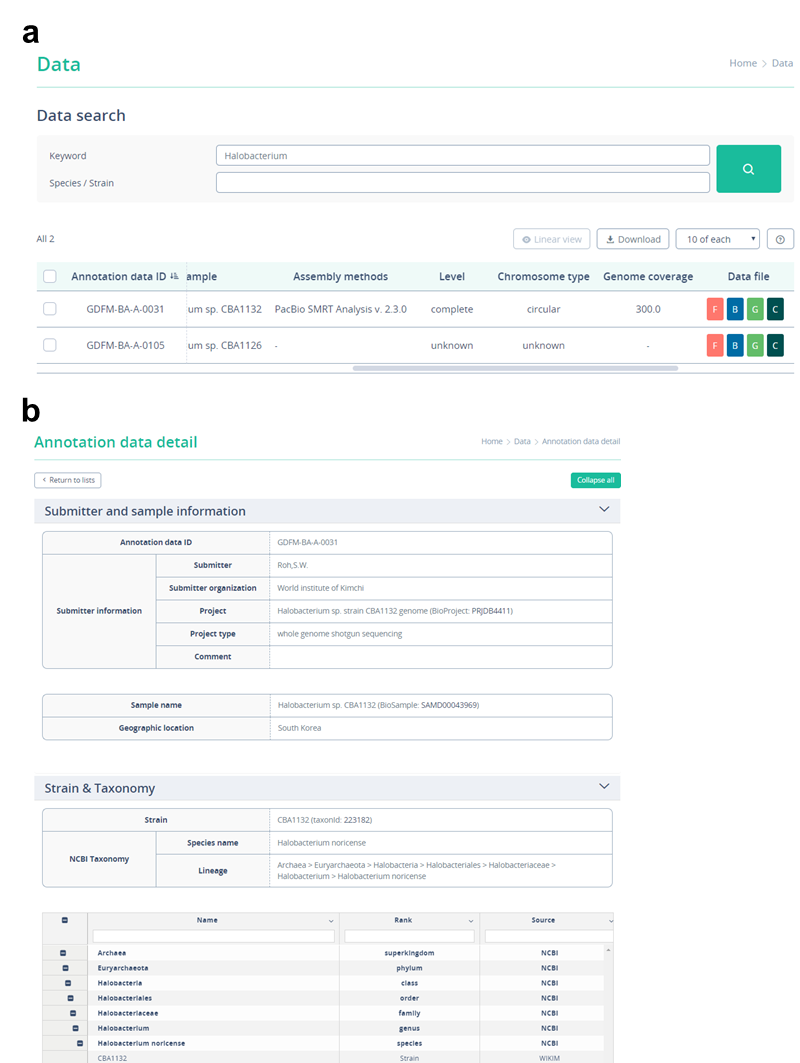


**Supplementary Fig. S2. Search results in the ODFM knowledgebase.** ODFM users can use either simple search or lexical search options. **a** In the data search page, the search results are presented in a tabular format, with each row depicting a microbial taxon. **b** In the annotation data detail page, the split function in each row shows detailed information regarding the annotation results. The annotation results presented in columns link to available datasets.

**
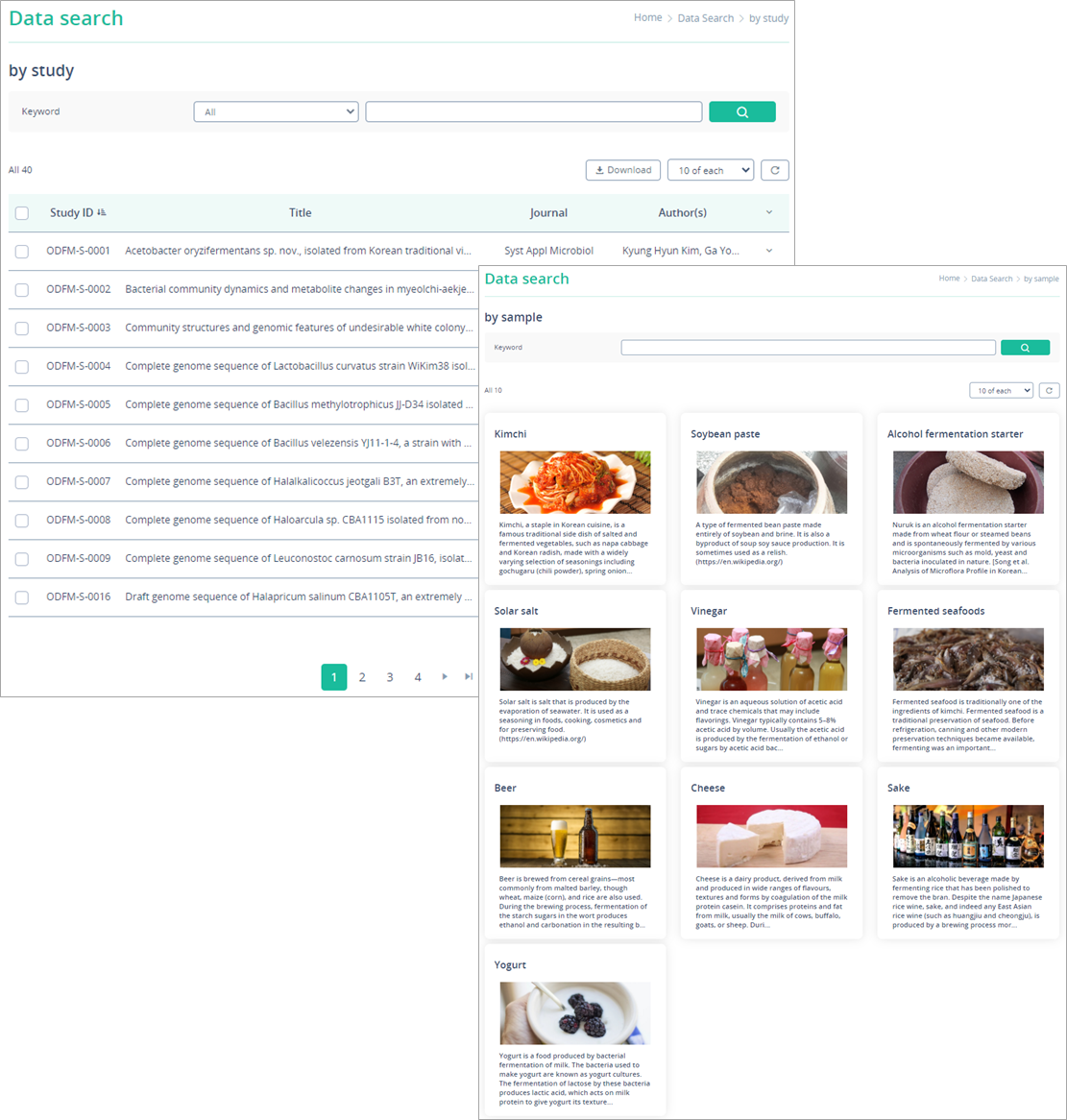
**

**Supplementary Fig. S3. Screen image of our projects tab in the ODFM.** Our projects tab on the front page provides a list of fermentation-associated microbial studies. The studies presented in columns link to detailed pages providing general information on the studies.

**Supplementary Table S1.** Detailed omics information of bacteria, archaea, and eukaryotic microorganisms in the ODFM.

| **No.** | **Data type** | **Taxonomy** | **Scientific name** | **Strain** | **Completeness** | **File type** | **Data availability** | **Sample (isolated source)** | **Assembly method** | **Study** |
| --- | --- | --- | --- | --- | --- | --- | --- | --- | --- | --- |
| 1 | Genome | Bacteria | *Lactobacillus allii* | WiKim39^T^ | Complete | Fasta | CP019323 | Kimchi | HGAP v. 3.0 | 1 |
| 2 | Genome | Bacteria | *Lactobacillus brevis* | WIKIM12 | Draft | Fasta | QQPA00000000 | Kimchi | CLC Genomics Workbench v. 6.5.1 | - |
| 3 | Genome | Bacteria | *Lactobacillus coryniformis* subsp*. corynifomis* | CBA3616 | Complete | Fasta | CP042392 | Kimchi | HGAP v. 3.0 | - |
| 4 | Genome | Bacteria | *Lactobacillus curvatus* | CBA3617 | Complete | Fasta | CP042389 | Kimchi | HGAP v. 3.0 | - |
| 5 | Genome | Bacteria | *Lactobacillus curvatus* | WiKim38 | Complete | Fasta | CP017124 | Kimchi | HGAP v. 3.0 | 2 |
| 6 | Genome | Bacteria | *Lactobacillus curvatus* | WiKim52 | Complete | Fasta | CP016602 | Kimchi | Canu v. 1.0 | - |
| 7 | Genome | Bacteria | *Lactobacillus malefermentans* | CBA3618 | Complete | Fasta | CP042371 | Kimchi | HGAP v. 3.0 | - |
| 8 | Genome | Bacteria | *Lactobacillus sakei* | CBA3614 | Complete | Fasta | CP046037 | Kimchi | HGAP v. 3.0 | - |
| 9 | Genome | Bacteria | *Lactobacillus sakei* | wikim 22 | Draft | Fasta | JRFY00000000 | Kimchi | CLC NGS Cell v. 7.0.4 | 3 |
| 10 | Genome | Bacteria | *Lactobacillus sakei* | WiKim0063 | Complete | Fasta | CP022709 | Kimchi | HGAP v. 3.0 | - |
| 11 | Genome | Bacteria | *Lactobacillus sakei* | WiKim0072 | Complete | Fasta | CP025136 | Kimchi | HGAP v. 3.0 | - |
| 12 | Genome | Bacteria | *Lactobacillus sakei* | WiKim0073 | Complete | Fasta | CP025203 | Kimchi | HGAP v. 3.0 | - |
| 13 | Genome | Bacteria | *Lactobacillus sakei* | WiKim0074 | Complete | Fasta | CP025206 | Kimchi | HGAP v. 3.0 | - |
| 14 | Genome | Bacteria | *Lactobacillus* sp. | CBA3605 | Complete | Fasta | CP027190 | Kimchi | HGAP v. 3.0 | - |
| 15 | Genome | Bacteria | *Lactobacillus* sp. | CBA3606 | Complete | Fasta | CP027194 | Kimchi | HGAP v. 3.0 | - |
| 16 | Genome | Bacteria | *Lactococcus lactis* subsp. *cremoris* | CBA3619 | Complete | Fasta | CP042408 | Kimchi | HGAP v. 3.0 | - |
| 17 | Genome | Bacteria | *Lactococcus raffinolactis* | WiKim0068 | Complete | Fasta | CP023392 | Kimchi | HGAP v. 3.0 | 4 |
| 18 | Genome | Bacteria | *Leuconostoc carnosum* | CBA3620 | Complete | Fasta | CP042374 | Kimchi | HGAP v. 3.0 | - |
| 19 | Genome | Bacteria | *Leuconostoc carnosum* | JB16 | Complete | Fasta | CP003851 | Kimchi | - | 5 |
| 20 | Genome | Bacteria | *Leuconostoc citreum* | CBA3621 | Complete | Fasta | CP042410 | Kimchi | HGAP v. 3.0 | - |
| 21 | Genome | Bacteria | *Leuconostoc citreum* | CBA3623 | Complete | Fasta | CP042393 | Kimchi | HGAP v. 3.0 | - |
| 22 | Genome | Bacteria | *Leuconostoc citreum* | CBA3624 | Complete | Fasta | CP042413 | Kimchi | HGAP v. 3.0 | - |
| 23 | Genome | Bacteria | *Leuconostoc citreum* | CBA3627 | Complete | Fasta | CP042418 | Kimchi | HGAP v. 3.0 | - |
| 24 | Genome | Bacteria | *Leuconostoc citreum* | WiKim0101 | Draft | Fasta | CP046149 | Kimchi | HGAP v. 3.0 | - |
| 25 | Genome | Bacteria | *Leuconostoc gelidum* | JB7 | Complete | Fasta | CP003839 | Kimchi | - | 6 |
| 26 | Genome | Bacteria | *Leuconostoc lactis* | CBA3622 | Complete | Fasta | CP042420 | Kimchi | HGAP v. 3.0 | - |
| 27 | Genome | Bacteria | *Leuconostoc lactis* | CBA3625 | Complete | Fasta | CP042387 | Kimchi | HGAP v. 3.0 | - |
| 28 | Genome | Bacteria | *Leuconostoc lactis* | CBA3626 | Complete | Fasta | CP042390 | Kimchi | HGAP v. 3.0 | - |
| 29 | Genome | Bacteria | *Leuconostoc lactis* | KACC 91922 | Draft | Fasta | JMEA01000000 | Kimchi | CLC Genomics Workbench v. 6.5 | 7 |
| 30 | Genome | Bacteria | *Leuconostoc lactis* | WIKIM21 | Draft | Fasta | LJIV00000000 | Kimchi | GS De Novo Assembler v. 2.9 | - |
| 31 | Genome | Bacteria | *Leuconostoc lactis* | WiKim40 | Complete | Fasta | CP016598 | Kimchi | Canu v. 1.3 | - |
| 32 | Genome | Bacteria | *Leuconostoc mesenteroides* | WiKim17 | Draft | Fasta | BBPK00000000 | Kimchi | CLC Genomics Workbench v. 7.0.4 | 8 |
| 33 | Genome | Bacteria | *Leuconostoc mesenteroides* | WiKim33 | Complete | Fasta | CP021491 | Kimchi | HGAP v. 3.0 | - |
| 34 | Genome | Bacteria | *Leuconostoc mesenteroides* subsp*. dextranicum* | CBA3628 | Complete | Fasta | CP042404 | Kimchi | HGAP v. 3.0 | - |
| 35 | Genome | Bacteria | *Leuconostoc mesenteroides* subsp. *jonggajibkimchii* | DRC 1506^T^ | Complete | Fasta | CP014611 | Kimchi | Canu v. 1.1 | 8 |
| 36 | Genome | Bacteria | *Leuconostoc mesenteroides* subsp*. mesenteroides* | CBA3607 | Complete | Fasta | CP046062 | Kimchi | HGAP v. 3.0 | - |
| 37 | Genome | Bacteria | *Leuconostoc mesenteroides* subsp. *mesenteroides* | DRC0211 | Complete | Fasta | CP013016 | Kimchi | HGAP v. 3.0 | 8 |
| 38 | Genome | Bacteria | *Leuconostoc mesenteroides* subsp. *mesenteroides* | J18 | Complete | Fasta | CP003101 | Kimchi | - | 8 |
| 39 | Genome | Bacteria | *Leuconostoc pseudomesenteroides* | CBA3630 | Complete | Fasta | CP042383 | Kimchi | HGAP v. 3.0 | - |
| 40 | Genome | Bacteria | *Leuconostoc* sp. | C2 | Complete | Fasta | CP002898 | Kimchi | - | 9 |
| 41 | Genome | Bacteria | *Leuconostoc suionicum* | DSM 20241^T^ | Complete | Fasta | CP015247 | Kimchi | HGAP v. 3.0 | 10 |
| 42 | Genome | Bacteria | *Oceanobacillus* *kimchii* | X50^T^ | Complete | Fasta | CM001792 | Kimchi | GS De Novo Assembler v. 2.6;  CLC NGS Cell v. 5.0 | 11 |
| 43 | Genome | Bacteria | *Pistricoccus aurantiacus* | CBA4606 | Complete | Fasta | CP042382 | Kimchi | HGAP v. 3.0 | - |
| 44 | Genome | Bacteria | *Weissella cibaria* | CBA3612 | Complete | Fasta | CP041193 | Kimchi | HGAP v. 3.0 | - |
| 45 | Genome | Bacteria | *Weissella hellenica* | CBA3632 | Complete | Fasta | CP042399 | Kimchi | HGAP v. 3.0 | - |
| 46 | Genome | Bacteria | *Weissella hellenica* | wikim14 | Draft | Fasta | BBIK00000000 | Kimchi | CLC Genomics Workbench v. 7.0.4 | - |
| 47 | Genome | Bacteria | *Weissella koreensis* | CBA3615 | Draft | Fasta | CP046070 | Kimchi | HGAP v. 3.0 | - |
| 48 | Genome | Bacteria | *Weissella koreensis* | KACC 15510 | Complete | Fasta | CP002899 | Kimchi | - | 12 |
| 49 | Genome | Bacteria | *Weissella koreensis* | WiKim0080 | Complete | Fasta | CP026847 | Kimchi | HGAP v. 3.0 | 13 |
| 50 | Genome | Bacteria | *Weissella soli* | CBA3633 | Draft | Fasta | CP042377 | Kimchi | HGAP v. 3.0 | - |
| 51 | Genome | Bacteria | *Bacillus velezensis* | JJ-D34 | Complete | Fasta | CP011346 | Soybean paste | CLC Genomics Workbench v. 7.5.1 | 14 |
| 52 | Genome | Bacteria | *Bacillus velezensis* | YJ11-1-4 | Complete | Fasta | CP011347 | Soybean paste | HGAP v. 2.0 | 15 |
| 53 | Genome | Bacteria | *Corynebacterium* *nuruki* | S6-4^T^ | Draft | Fasta | AFIZ00000000 | Alcohol fermentation starter | GS De Novo Assembler v. 2.5.3 | 16 |
| 54 | Genome | Bacteria | *Acetobacter oryzifermentans* | SLV-7^T^ | Complete | Fasta | CP011120 | Vinegar | ICORN v. unspecified | 17 |
| 55 | Genome | Bacteria | *Acetobacter pasteurianus* subsp. *ascendens* | LMG 1590^T^ | Complete | Fasta | CP015164 | Vinegar | FALCON v. MARCH-2016 | 18 |
| 56 | Genome | Bacteria | *Acetobacter pasteurianus* subsp. *paradoxus* | LMG 1591^T^ | Complete | Fasta | CP015168 | Vinegar | FALCON v. MARCH-2016 | 18 |
| 57 | Genome | Bacteria | *Alishewanella jeotgali* | KCTC 22429 | Draft | Fasta | AHTH01000000 | Fermented seafood | CLC Genomics Workbench v. 4.7.2;  GS De Novo Assembler v. 2.5.1 | 19 |
| 58 | Genome | Bacteria | *Brachybacterium* *squillarum* | M-6-3^T^ | Draft | Fasta | AGBX00000000 | Fermented seafood | CLC Genomics Workbench v. 4.2;  GS De Novo Assembler v. 2.5 | 20 |
| 59 | Genome | Bacteria | *Dietzia* *alimentaria* | 72^T^ | Draft | Fasta | AGFF00000000 | Fermented seafood | CLC Genomics Workbench v. 4.2;  GS De Novo Assembler v. 2.5 | 21 |
| 60 | Genome | Bacteria | *Lentibacillus* *jeotgali* | Grbi^T^ | Draft | Fasta | AGAV00000000 | Fermented seafood | CLC Genomics Workbench v. 4.0;  GS De Novo Assembler v. 2.5 | 22 |
| 61 | Genome | Bacteria | *Microbacterium* sp*.* | CBA3102 | Draft | Fasta | CP042412 | Fermented seafood | HGAP v. 3.0 | - |
| 62 | Genome | Bacteria | *Paracoccus jeotgali* | CBA4604^T^ | Complete | Fasta | CP025583 | Fermented seafood | HGAP v. 3.0 | 23 |
| 63 | Genome | Bacteria | *Salimicrobium jeotgali* | MJ3^T^ | Complete | Fasta | CP011361 | Fermented seafood | HGAP v. 3.0 | 24 |
| 64 | Genome | Bacteria | *Staphylococcus* sp. | OJ82 | Draft | Fasta | ALPU01000000 | Fermented seafood | GS De Novo Assembler v. 2.6 | 25 |
| 65 | Genome | Bacteria | *Tetragenococcus halophilus* | MJ4 | Complete | Fasta | CP012047 | Fermented seafood | HGAP v. 3.0 | 26 |
| 66 | Genome | Bacteria | *Acetobacter cerevisiae* | LMG 1625 | Draft | Fasta | GCA_001580535.1 | Beer | CLC Genomics Workbench v. 7.0 | 27 |
| 67 | Genome | Bacteria | *Acetobacter malorum* | LMG 1604 | Draft | Fasta | GCA_001581085.1 | Beer | CLC Genomics Workbench v. 7.0 | - |
| 68 | Genome | Bacteria | *Acetobacter orleanensis* | LMG 1583 | Draft | Fasta | GCA_001581005.1 | Beer | CLC Genomics Workbench v. 7.0 | 28 |
| 69 | Genome | Bacteria | *Acetobacter pasteurianus* | BCRC14145 | Draft | Fasta | GCA_003332155.1 | Beer | MIRA v. 3.4 | 29 |
| 70 | Genome | Bacteria | *Lactobacillus amylolyticus* | LA664 | Complete | Fasta | CP045584 | Beer | HGAP v. 2.0/3.0 | - |
| 71 | Genome | Bacteria | *Lactobacillus backii* | JCM 18665 | Draft | Fasta | GCA_003946275.1 | Beer | A5-MiSeq v. 20150522 | 30 |
| 72 | Genome | Bacteria | *Lactobacillus brevis* | TMW 1.2111 | Complete | Fasta | CP019743 | Beer | Celera Assembler v. 1 | 31 |
| 73 | Genome | Bacteria | *Lactobacillus cerevisiae* | DSM 100836 | Draft | Fasta | GCA_003946245.1 | Beer | A5-MiSeq v. 20150522 | 32 |
| 74 | Genome | Bacteria | *Lactobacillus lindneri* | TMW 1.2082 | Draft | Fasta | GCA_002907075.1 | Beer | SPAdes v. 3.9.0 | - |
| 75 | Genome | Bacteria | *Lactobacillus paracasei* | CCC B1205 | Draft | Fasta | GCA_002904305.1 | Beer | A5 Pipeline v. April-2014 | - |
| 76 | Genome | Bacteria | *Lactobacillus paracollinoides* | TMW 1.1995 | Complete | Fasta | CP014924 | Beer | Celera Assembler v. 1 | 33 |
| 77 | Genome | Bacteria | *Lactobacillus paraplantarum* | DSM 10667 | Complete | Fasta | CP032744 | Beer | HGAP v. 2.0/3.0 | - |
| 78 | Genome | Bacteria | *Megasphaera cerevisiae* | NSB1 | Draft | Fasta | GCA_001913815.1 | Beer | SPAdes v. 3.9.0 | 34 |
| 79 | Genome | Bacteria | *Pectinatus frisingensis* | MB139 | Draft | Fasta | GCA_014076555.1 | Beer | HGAP v. 2.0/3.0 | 35 |
| 80 | Genome | Bacteria | *Pediococcus acidilactici* | BIO6314 | Draft | Fasta | GCA_013249135.1 | Beer | SPAdes v. 3.13.1 | - |
| 81 | Genome | Bacteria | *Pediococcus claussenii* | TMW 2.54 | Complete | Fasta | CP014936 | Beer | Celera Assembler v. 1 | 36 |
| 82 | Genome | Bacteria | *Pediococcus damnosus* | VTT E-123216 | Draft | Fasta | GCA_002770635.1 | Beer | SPAdes v. 3.9.0 | 37 |
| 83 | Genome | Bacteria | *Brachybacterium alimentarium* | 962_10 | Draft | Fasta | GCA_003335295.1 | Cheese | Canu v. 1.5 | - |
| 84 | Genome | Bacteria | *Brevibacterium aurantiacum* | SMQ-1420 | Complete | Fasta | GCA_003999315.1 | Cheese | Celera Assembler v. 8.3 | - |
| 85 | Genome | Bacteria | *Clostridium botulinum* | CDC66089 | Draft | Fasta | GCA_000730865.1 | Cheese | MIRA v. 3.9.9 | - |
| 86 | Genome | Bacteria | *Clostridium tyrobutyricum* | FAM22553 | Draft | Fasta | GCA_000816635.1 | Cheese | SPAdes v. 3.1.0 | 38 |
| 87 | Genome | Bacteria | *Corynebacterium flavescens* | CCUG 28791^T^ | Draft | Fasta | GCA_008693105.1 | Cheese | SPAdes v. 3.1.0 | 39 |
| 88 | Genome | Bacteria | *Enterococcus durans* | IQ23 | Draft | Fasta | GCA_001455455.1 | Cheese | A5 pipeline v. FEB-2015 | 40 |
| 89 | Genome | Bacteria | *Enterococcus faecalis* | DM7-2 | Draft | Fasta | GCA_002108195.1 | Cheese | SOAPdenovo v. 2.0 | - |
| 90 | Genome | Bacteria | *Enterococcus faecium* | IQ110 | Draft | Fasta | GCA_001455445.1 | Cheese | SeqMan NGen v. 12 | 40 |
| 91 | Genome | Bacteria | *Enterococcus lactis* | CICC 24101 | Draft | Fasta | GCA_009735495.1 | Cheese | SPAdes v. 3.11.0 | - |
| 92 | Genome | Bacteria | *Enterococcus malodoratus* | DSM 20681 | Draft | Fasta | GCA_001886025.1 | Cheese | SOAPdenovo v. 2.0 | 41 |
| 93 | Genome | Bacteria | *Escherichia coli* | FAM21805 | Draft | Fasta | GCA_002109485.1 | Cheese | CLC Genomics Workbench v. 9.0.1 | - |
| 94 | Genome | Bacteria | *Glutamicibacter arilaitensis* | JB182 | Draft | Fasta | GCA_002878675.1 | Cheese | Canu v. 1.5 | 42 |
| 95 | Genome | Bacteria | *Hafnia alvei* | B16-3 | Draft | Fasta | GCA_012689215.1 | Cheese | SPAdes v. 3.11.1 | - |
| 96 | Genome | Bacteria | *Halomonas nigrificans* | MBT G8648 | Draft | Fasta | GCA_002374315.1 | Cheese | SPAdes v. 3.10.1 | 43 |
| 97 | Genome | Bacteria | *Lactobacillus acidophilus* | KLDS 1.0901 | Draft | Fasta | GCA_001868765.1 | Cheese | SOAPdenovo v. 2.4 | - |
| 98 | Genome | Bacteria | *Lactobacillus bifermentans* | LB003 | Complete | Fasta | CP045872 | Cheese | HGAP v. 2.0/3.0 | 44 |
| 99 | Genome | Bacteria | *Lactobacillus brevis* | D6 | Draft | Fasta | GCA_001541605.1 | Cheese | Newbler v. 2.5.3 | 45 |
| 100 | Genome | Bacteria | *Lactobacillus casei* | GCRL 163 | Draft | Fasta | GCA_002091995.1 | Cheese | ABySS v. 1.9.0 | 46 |
| 101 | Genome | Bacteria | *Lactobacillus helveticus* | NWC_2_4 | Draft | Fasta | GCA_003814325.1 | Cheese | Flye v. 2.3.3 | 47 |
| 102 | Genome | Bacteria | *Lactobacillus parabuchneri* | IPLA 11117 | Draft | Fasta | GCA_001687155.1 | Cheese | SPAdes v. 3.6.2 | - |
| 103 | Genome | Bacteria | *Lactobacillus paracasei* | DPC 7150 | Draft | Fasta | GCA_014155825.1 | Cheese | SPAdes v. 3.7.0 | - |
| 104 | Genome | Bacteria | *Lactobacillus plantarum* | WHE 92 | Draft | Fasta | GCA_000604145.1 | Cheese | CLC NGS Cell v. 6.0.2 | 48 |
| 105 | Genome | Bacteria | *Lactobacillus rhamnosus* | DPC 7102 | Draft | Fasta | GCA_014155845.1 | Cheese | SPAdes v. 3.7.0 | - |
| 106 | Genome | Bacteria | *Lactococcus lactis* subsp*. cremoris* | LMG6897 | Draft | Fasta | GCA_001622295.1 | Cheese | IDBA-UD v. 1.1.1 | - |
| 107 | Genome | Bacteria | *Lactococcus lactis* subsp*. lactis* | CECT 4433 | Draft | Fasta | GCA_000761565.1 | Cheese | MIRA v. 3.9; CAP3 v. 1 | 49 |
| 108 | Genome | Bacteria | *Leuconostoc mesenteroides* subsp*. cremoris* | LbT16 | Draft | Fasta | GCA_001184265.1 | Cheese | SPAdes v. 3.5.0 | 50 |
| 109 | Genome | Bacteria | *Leuconostoc mesenteroides* subsp*. dextranicum* | DSM 20484 | Complete | Fasta | CP012009 | Cheese | HGAP v. 2.0 | 51 |
| 110 | Genome | Bacteria | *Leuconostoc mesenteroides* subsp*. dextranicum* | LbE15 | Draft | Fasta | GCA_001184245.1 | Cheese | SPAdes v. 3.5.0 | - |
| 111 | Genome | Bacteria | *Leuconostoc mesenteroides* subsp*. mesenteroides* | LbE16 | Draft | Fasta | GCA_001184255.1 | Cheese | SPAdes v. 3.5.0 | - |
| 112 | Genome | Bacteria | *Listeria monocytogenes* | N11-2542 | Draft | Fasta | GCA_003589105.1 | Cheese | SPAdes v. 3.0 | - |
| 113 | Genome | Bacteria | *Morganella morganii* | FAM24091 | Draft | Fasta | GCA_003034205.1 | Cheese | SPAdes v. 3.11.1 | 52 |
| 114 | Genome | Bacteria | *Mycetocola reblochoni* | JCM 30549 | Draft | Fasta | GCA_003667525.1 | Cheese | SOAPdenovo v. 4.0 | 53 |
| 115 | Genome | Bacteria | *Propionibacterium freudenreichii* | FAM 14217 | Complete | Fasta | CP053853 | Cheese | HGAP v. 3.0 | - |
| 116 | Genome | Bacteria | *Pseudomonas fluorescens* | ITEM 17298 | Draft | Fasta | GCA_002319065.1 | Cheese | SPAdes v. 3.5.0 | 54 |
| 117 | Genome | Bacteria | *Pseudomonas lactis* | ITEM 17295 | Draft | Fasta | GCA_014062385.1 | Cheese | SPAdes v. 3.5.0 | 55 |
| 118 | Genome | Bacteria | *Staphylococcus aureus* | 18SBCL679 | Draft | Fasta | GCA_013343875.1 | Cheese | SPAdes v. 3.12.0 | - |
| 119 | Genome | Bacteria | *Staphylococcus equorum* | 876_5 | Draft | Fasta | GCA_004143695.1 | Cheese | CLC Genomics Workbench v. 8.0.1 | - |
| 120 | Genome | Bacteria | *Staphylococcus saprophyticus* | DPC5671 | Draft | Fasta | GCA_002009035.1 | Cheese | Newbler v. 2.3 | 56 |
| 121 | Genome | Bacteria | *Staphylococcus succinus* | BC15 | Draft | Fasta | GCA_001747705.1 | Cheese | CLC Genomics Workbench v. 8.0.1 | - |
| 122 | Genome | Bacteria | *Staphylococcus xylosus* | BC10 | Draft | Fasta | GCA_001747745.1 | Cheese | CLC Genomics Workbench v. 8.0.1 | - |
| 123 | Genome | Bacteria | *Streptococcus thermophilus* | C106 | Draft | Fasta | GCA_001306395.1 | Cheese | CLS Software Suite v. Sep-2013 | 57 |
| 124 | Genome | Bacteria | *Vibrio casei* | JB196 | Draft | Fasta | GCA_003335255.1 | Cheese | Canu v. 1.5 | 42 |
| 125 | Genome | Bacteria | *Lactobacillus acetotolerans* | NBRC 13120 | Complete | Fasta | AP014808 | Sake | Phrap v. 1.080730 | 58 |
| 126 | Genome | Bacteria | *Lactobacillus homohiochii* | DSM 20571 | Draft | Fasta | GCA_001436985.1 | Sake | SOAPdenovo v. 2.0 | - |
| 127 | Genome | Bacteria | *Pediococcus pentosaceus* | KCCM 40703 | Complete | Fasta | CP020018 | Sake | HGAP v. 2.0 | 59 |
| 128 | Genome | Bacteria | *Enterococcus faecalis* | XJ76305 | Draft | Fasta | GCA_002107085.1 | Yogurt | SOAPdenovo v. 2.0 | 60 |
| 129 | Genome | Bacteria | *Lactobacillus acidophilus* | FSI4 | Complete | Fasta | GCA_000934625.1 | Yogurt | velvet v. 1.2.10;  Phred/Phrap/Consed v. 24 | 61 |
| 130 | Genome | Bacteria | *Lactobacillus delbrueckii* subsp*. bulgaricus* | CRL871 | Draft | Fasta | GCA_000934805.1 | Yogurt | SeqMan NGen v. 11.2.1.25 | 62 |
| 131 | Genome | Bacteria | *Streptococcus thermophilus* | MN-BM-A01 | Complete | Fasta | GCA_001280285.1 | Yogurt | HGAP v. 2.0/3.0 | 63 |
| 132 | Genome | Archaea | *Haladaptatus cibarius* | D43^T^ | Draft | Fasta | JDTH00000000 | Fermented seafood | CLC Genomics Workbench v. 6.5.1 | 64 |
| 133 | Genome | Archaea | *Halalkalicoccus jeotgali* | B3^T^ | Complete | Fasta | CP002062 | Fermented seafood | - | 65 |
| 134 | Genome | Archaea | *Halapricum salinum* | CBA1105^T^ | Draft | Fasta | BBMO00000000 | Solar salt | CLC Genomics Workbench v. 7.0.4 | 66 |
| 135 | Genome | Archaea | *Halapricum* sp. | CBA1109 | Draft | Fasta | WPCA00000000 | Solar salt | CLC Genomics Workbench v. 6.5.1 | - |
| 136 | Genome | Archaea | *Halarchaeum* sp. | CBA1220 | Draft | Fasta | RIZB00000000 | Solar salt | HGAP v. 2.0/3.0 | - |
| 137 | Genome | Archaea | *Haloarcula* *hispanica* | CBA1121 | Draft | Fasta | RQWK00000000 | Solar salt | HGAP v. 3.0 | - |
| 138 | Genome | Archaea | *Haloarcula hispanica* | CBA1128 | Draft | Fasta | BCNA00000000 | Solar salt | HGAP v. 2.0/3.0 | - |
| 139 | Genome | Archaea | *Haloarcula marismortui* | CBA1127 | Draft | Fasta | BCNB00000000 | Solar salt | HGAP v. 2.0/3.0 | - |
| 140 | Genome | Archaea | *Haloarcula* sp. | CBA1115 | Complete | Fasta | CP010529 | Solar salt | HGAP v. 3.0 | 67 |
| 141 | Genome | Archaea | *Haloarcula* sp*.* | CBA1122 | Draft | Fasta | WPCB00000000 | Solar salt | CLC Genomics Workbench v. 6.5.1 | - |
| 142 | Genome | Archaea | *Haloarcula* sp. | CBA1129 | Draft | Fasta | RKSM00000000 | Solar salt | HGAP v. 2.0/3.0 | - |
| 143 | Genome | Archaea | *Haloarcula* sp. | CBA1130 | Draft | Fasta | RPCX00000000 | Solar salt | HGAP v. 2.0/3.0 | - |
| 144 | Genome | Archaea | *Haloarcula* sp. | CBA1131 | Draft | Fasta | RPCY00000000 | Solar salt | HGAP v. 2.0/3.0 | - |
| 145 | Genome | Archaea | *Halobacterium noricense* | CBA1132 | Complete | Fasta | BCMZ00000000 | Solar salt | HGAP v. 2.0/3.0 | 68 |
| 146 | Genome | Archaea | *Halobacterium* sp. | CBA1126 | Draft | Fasta | WPCF00000000 | Solar salt | CLC Genomics Workbench v. 6.5.1 | - |
| 147 | Genome | Archaea | *Halobellus rufus* | CBA1103^T^ | Draft | Fasta | BBJO00000000 | Solar salt | CLC Genomics Workbench v. 6.5 | 69 |
| 148 | Genome | Archaea | *Halococcus sediminicola* | CBA1101^T^ | Draft | Fasta | BBMP00000000 | Solar salt | CLC Genomics Workbench v. 7.0.4 | 70 |
| 149 | Genome | Archaea | *Haloferax* sp. | CBA1148 | Draft | Fasta | VZUR00000000 | Solar salt | HGAP v. 2.0/3.0 | - |
| 150 | Genome | Archaea | *Haloferax* sp. | CBA1149 | Draft | Fasta | VZUS00000000 | Solar salt | HGAP v. 2.0/3.0 | - |
| 151 | Genome | Archaea | *Haloferax* sp. | CBA1150 | Draft | Fasta | VZUU00000000 | Solar salt | HGAP v. 2.0/3.0 | - |
| 152 | Genome | Archaea | *Halogeometricum* sp. | CBA1124 | Draft | Fasta | WPCD00000000 | Solar salt | CLC Genomics Workbench v. 6.5.1 | - |
| 153 | Genome | Archaea | *Halolamina rubra* | CBA1107^T^ | Draft | Fasta | BBJN00000000 | Solar salt | CLC Genomics Workbench v. 6.5 | 71 |
| 154 | Genome | Archaea | *Halolamina salina* | CBA1230 | Draft | Fasta | MWUK00000000 | Solar salt | HGAP v. 2.0/3.0 | - |
| 155 | Genome | Archaea | *Halopenitus persicus* | CBA1233 | Complete | Fasta | AP017558 | Solar salt | HGAP v. 2.0/3.0 | - |
| 156 | Genome | Archaea | *Haloplanus* sp. | CBA1112 | Complete | Fasta | CP031148 | Solar salt | HGAP v. 2.0/3.0 | - |
| 157 | Genome | Archaea | *Haloplanus* sp. | CBA1113 | Complete | Fasta | CP031150 | Solar salt | HGAP v. 2.0/3.0 | - |
| 158 | Genome | Archaea | *Halorhabdus* sp. | CBA1104 | Complete | Fasta | CP033878 | Solar salt | HGAP v. 3.0 | - |
| 159 | Genome | Archaea | *Halorubrum halophilum* | B8^T^ | Draft | Fasta | BBJP00000000 | Fermented seafood | CLC Genomics Workbench v. 7.0.4 | 72 |
| 160 | Genome | Archaea | *Halorubrum halophilum* | CBA1147 | Draft | Fasta | VZUT00000000 | Fermented seafood | HGAP v. 2.0/3.0 | - |
| 161 | Genome | Archaea | *Halorubrum* sp*.* | CBA1125 | Draft | Fasta | WPCE00000000 | Solar salt | CLC Genomics Workbench v. 6.5.1 | - |
| 162 | Genome | Archaea | *Halorubrum* sp*.* | CBA1229 | Draft | Fasta | RIZC00000000 | Solar salt | HGAP v. 2.0/3.0 | - |
| 163 | Genome | Archaea | *Halorubrum* sp. | CBA1232 | Draft | Fasta | AP017569 | Solar salt | HGAP v. 2.0/3.0 | - |
| 164 | Genome | Archaea | *Halostella salina* | CBA1114 | Draft | Fasta | RCIH00000000 | Solar salt | SOAPdenovo v. 2.0 | - |
| 165 | Genome | Archaea | *Haloterrigena jeotgali* | A29^T^ | Draft | Fasta | JDTG00000000 | Fermented seafood | CLC Genomics Workbench v. 6.5.1 | 73 |
| 166 | Genome | Archaea | *Natrinema* sp. | CBA1119^T^ | Draft | Fasta | PDBS00000000 | Solar salt | HGAP v. 2.0/3.0 | 74 |
| 167 | Genome | Archaea | *Natronomonas* sp*.* | CBA1123 | Draft | Fasta | WPCC00000000 | Solar salt | CLC Genomics Workbench v. 6.5.1 | - |
| 168 | Genome | Archaea | *Natronomonas* sp. | CBA1133 | Draft | Fasta | RJJC00000000 | Solar salt | HGAP v. 2.0/3.0 | - |
| 169 | Genome | Archaea | *Natronomonas* sp. | CBA1134 | Draft | Fasta | BDJH00000000 | Solar salt | HGAP v. 2.0/3.0 | - |
| 170 | Genome | Eukarya | *Candida sake* | CBA6005 | Draft | Fasta | QELA00000000 | Kimchi | HGAP v. 4.0 | 75 |
| 171 | Genome | Eukarya | *Hanseniaspora uvarum* | CBA6001 | Draft | Fasta | PTQS00000000 | Kimchi | HGAP v. 4.0 | 75 |
| 172 | Genome | Eukarya | *Kazachstania servazzii* | CBA6004 | Draft | Fasta | PTQT00000000 | Kimchi | HGAP v. 4.0 | 75 |
| 173 | Genome | Eukarya | *Pichia kluyveri* | CBA6002 | Draft | Fasta | QEFR00000000 | Kimchi | HGAP v. 4.0 | 75 |
| 174 | Genome | Eukarya | *Yarrowia lipolytica* | CBA6003 | Draft | Fasta | QFFT00000000 | Kimchi | HGAP v. 4.0 | 75 |
| 175 | Genome | Eukarya | *Brettanomyces anomalus* | YV396 | Draft | Fasta | LCTY00000000 | Beer | SOAPdenovo v. 1.05 | - |
| 176 | Genome | Eukarya | *Saccharomyces cerevisiae* | beer007 | Complete | Fasta | MBZV00000000 | Beer | idba_ud v. 1.1.1 | 76 |
| 177 | Genome | Eukarya | *Saccharomyces cerevisiae* | beer009 | Complete | Fasta | MBZT00000000 | Beer | idba_ud v. 1.1.1 | 76 |
| 178 | Genome | Eukarya | *Saccharomyces cerevisiae* | beer011 | Complete | Fasta | MBZR00000000 | Beer | idba_ud v. 1.1.1 | 76 |
| 179 | Genome | Eukarya | *Saccharomyces cerevisiae* | beer012 | Complete | Fasta | MBZQ00000000 | Beer | idba_ud v. 1.1.1 | 76 |
| 180 | Genome | Eukarya | *Saccharomyces cerevisiae* | beer018 | Complete | Fasta | MBZK00000000 | Beer | idba_ud v. 1.1.1 | 76 |
| 181 | Genome | Eukarya | *Saccharomyces cerevisiae* | beer024 | Complete | Fasta | MBZE00000000 | Beer | idba_ud v. 1.1.1 | 76 |
| 182 | Genome | Eukarya | *Saccharomyces cerevisiae* | beer031 | Complete | Fasta | MBYX00000000 | Beer | idba_ud v. 1.1.1 | 76 |
| 183 | Genome | Eukarya | *Saccharomyces cerevisiae* | beer033 | Complete | Fasta | MBYV00000000 | Beer | idba_ud v. 1.1.1 | 76 |
| 184 | Genome | Eukarya | *Saccharomyces cerevisiae* | beer034 | Complete | Fasta | MBYU00000000 | Beer | idba_ud v. 1.1.1 | 76 |
| 185 | Genome | Eukarya | *Saccharomyces cerevisiae* | beer042 | Complete | Fasta | MBYM00000000 | Beer | idba_ud v. 1.1.1 | 76 |
| 186 | Genome | Eukarya | *Saccharomyces cerevisiae* | beer044 | Complete | Fasta | MBYK00000000 | Beer | idba_ud v. 1.1.1 | 76 |
| 187 | Genome | Eukarya | *Saccharomyces cerevisiae* | beer072 | Complete | Fasta | MBXI00000000 | Beer | idba_ud v. 1.1.1 | 76 |
| 188 | Genome | Eukarya | *Saccharomyces cerevisiae* | beer076 | Complete | Fasta | MBXE00000000 | Beer | idba_ud v. 1.1.1 | 76 |
| 189 | Genome | Eukarya | *Saccharomyces pastorianus* | CBS 1483 | Complete | Fasta | CP048983 | Beer | Canu v. 1.4 | 77 |
| 190 | Genome | Eukarya | *Saccharomyces pastorianus* | CBS 1513 | Draft | Fasta | AYZZ00000000 | Beer | Newbler v. 2.6 | 78 |
| 191 | Genome | Eukarya | *Saccharomyces pastorianus* | CBS 2440 | Draft | Fasta | DUYC00000000 | Beer | Platanus v. 1.2.4 | 79 |
| 192 | Genome | Eukarya | *Saccharomyces pastorianus* | DBVPG 6033i | Draft | Fasta | DUXV00000000 | Beer | SPAdes v. 3.9 | 80 |
| 193 | Genome | Eukarya | *Saccharomyces pastorianus* | HA2560 | Draft | Fasta | PQMD00000000 | Beer | Newbler v. 2.9 | 81 |
| 194 | Genome | Eukarya | *Saccharomyces pastorianus* | W34/70 | Draft | Fasta | DUWY00000000 | Beer | SPAdes v. 3.9 | 82 |
| 195 | Genome | Eukarya | *Saccharomyces pastorianus* | Weihenstephan 34/70 | Draft | Fasta | BBYY00000000 | Beer | Platanus v. 1.2.4 | 83 |
| 196 | Genome | Eukarya | *Penicillium roqueforti* | CECT 2905 | Draft | Fasta | GCA_001939915.1 | Cheese | Bowtie v. 1.1.1 | - |
| 197 | Genome | Eukarya | *Saccharomyces cerevisiae* | K11 | Draft | Fasta | GCA_000767965.1 | Sake | ABySS v. 1.3.5; SGA v. 0.9.35 | - |
| 198 | Metataxonome | Bacteria | - | - | - | Fasta | SRA044882 | Kimchi | - | 84 |
| 199 | Metataxonome | Bacteria | - | - | - | Fasta | SRA058753 | Kimchi | - | 85 |
| 200 | Metataxonome | Bacteria | - | - | - | Fasta | SRA055979 | Kimchi | - | 86 |
| 201 | Metataxonome | Bacteria | - | - | - | Fasta | SRA059491 | Kimchi | - | 87 |
| 202 | Metataxonome | Bacteria | - | - | - | Fasta | SRP044312 | Kimchi | - | 88 |
| 203 | Metataxonome | Bacteria | - | - | - | Fasta | SRP115161 | Kimchi | - | 89 |
| 204 | Metataxonome | Bacteria | - | - | - | Fasta | SRA050204 | Kimchi | - | 90 |
| 205 | Metataxonome | Bacteria | - | - | - | Fasta | SRP221859 | Kimchi | - | 91 |
| 206 | Metataxonome | Bacteria | - | - | - | Fasta | SRP039098 | Soybean paste | - | 92 |
| 207 | Metataxonome | Bacteria | - | - | - | Fasta | SRP072427 | Soybean paste | - | 93 |
| 208 | Metataxonome | Bacteria | - | - | - | Fasta | SRP184626 | Soybean paste | - | 94 |
| 209 | Metataxonome | Bacteria | - | - | - | Fasta | SRP049639 | Fermented seafood | - | 95 |
| 210 | Metataxonome | Bacteria | - | - | - | Fasta | SRP064726 | Fermented seafood | - | 96 |
| 211 | Metataxonome | Bacteria | - | - | - | Fasta | SRA058343 | Fermented seafood | - | 97 |
| 212 | Metataxonome | Bacteria | - | - | - | Fasta | SRA067250 | Fermented seafood | - | 98 |
| 213 | Metataxonome | Bacteria | - | - | - | Fasta | SRS474925 | Fermented seafood | - | 99 |
| 214 | Metataxonome | Bacteria | - | - | - | Fasta | SRP111432 | Fermented seafood | - | 21 |
| 215 | Metagenome | Viruses | - | - | - | Fastq | PRJEB23957 | Kimchi | - | 100 |
| 216 | Metagenome | - | - | - | - | Fasta | SRA023444 | Kimchi | - | 101 |
| 217 | Metatranscriptome | - | - | - | - | Fastq | SRA050204 | Kimchi | - | 90 |
| 218 | Transcriptome | Archaea | - | - | - | Fastq | - | Solar salt | - | - |
| 219 | Metabolome | - | - | - | - | Excel | - | Kimchi | - | 85 |
| 220 | Metabolome | - | - | - | - | Excel | - | Kimchi | - | 89 |
| 221 | Metabolome | - | - | - | - | Excel | - | Kimchi | - | 91 |
| 222 | Metabolome | - | - | - | - | Excel | - | Fermented seafood | - | 98 |
| 223 | Metabolome | - | - | - | - | Excel | - | Fermented seafood | - | 96 |
| 224 | Metabolome | - | - | - | - | Excel | - | Fermented seafood | - | 95 |
| 225 | Metabolome | - | - | - | - | Excel | - | Fermented seafood | - | 26 |

**References**

1 Jung, M. Y., Lee, S. H., Lee, M., Song, J. H. & Chang, J. Y. *Lactobacillus allii* sp. nov. isolated from scallion kimchi. *Int J Syst Evol Microbiol* **67**, 4936-4942, doi:10.1099/ijsem.0.002327 (2017).

2 Lee, S. H., Jung, M. Y., Song, J. H., Lee, M. & Chang, J. Y. Complete Genome Sequence of *Lactobacillus curvatus* Strain WiKim38 Isolated from Kimchi. *Genome Announc* **5**, doi:10.1128/genomeA.00273-17 (2017).

3 Lim, H. I. *et al.* Draft Genome Sequence of *Lactobacillus sakei* Strain wikim 22, Isolated from Kimchi in Chungcheong Province, South Korea. *Genome Announc* **2**, doi:10.1128/genomeA.01296-14 (2014).

4 Jung, M. Y., Lee, C., Seo, M. J., Roh, S. W. & Lee, S. H. Characterization of a potential probiotic bacterium *Lactococcus raffinolactis* WiKim0068 isolated from fermented vegetable using genomic and in vitro analyses. *BMC Microbiol* **20**, 136, doi:10.1186/s12866-020-01820-9 (2020).

5 Jung, J. Y., Lee, S. H. & Jeon, C. O. Complete genome sequence of *Leuconostoc carnosum* strain JB16, isolated from kimchi. *J Bacteriol* **194**, 6672-6673, doi:10.1128/JB.01805-12 (2012).

6 Jung, J. Y., Lee, S. H. & Jeon, C. O. Complete genome sequence of *Leuconostoc gelidum* strain JB7, isolated from kimchi. *J Bacteriol* **194**, 6665, doi:10.1128/JB.01806-12 (2012).

7 Moon, J. S. *et al.* Genome sequence analysis of potential probiotic strain *Leuconostoc lactis* EFEL005 isolated from kimchi. *J Microbiol* **53**, 337-342, doi:10.1007/s12275-015-5090-8 (2015).

8 Chun, B. H., Kim, K. H., Jeon, H. H., Lee, S. H. & Jeon, C. O. Pan-genomic and transcriptomic analyses of *Leuconostoc mesenteroides* provide insights into its genomic and metabolic features and roles in kimchi fermentation. *Sci Rep* **7**, 11504, doi:10.1038/s41598-017-12016-z (2017).

9 Lee, S. H., Jung, J. Y., Lee, S. H. & Jeon, C. O. Complete genome sequence of *Leuconostoc kimchii* strain C2, isolated from Kimchi. *J Bacteriol* **193**, 5548, doi:10.1128/JB.05707-11 (2011).

10 Chun, B. H., Lee, S. H., Jeon, H. H., Kim, D. W. & Jeon, C. O. Complete genome sequence of *Leuconostoc suionicum* DSM 20241^T^ provides insights into its functional and metabolic features. *Stand Genomic Sci* **12**, 38, doi:10.1186/s40793-017-0256-0 (2017).

11 Whon, T. W. *et al.* *Oceanobacillus kimchii* sp. nov. isolated from a traditional Korean fermented food. *J Microbiol* **48**, 862-866, doi:10.1007/s12275-010-0214-7 (2010).

12 Lee, S. H., Jung, J. Y., Lee, S. H. & Jeon, C. O. Complete genome sequence of *Weissella koreensis* KACC 15510, isolated from kimchi. *J Bacteriol* **193**, 5534, doi:10.1128/JB.05704-11 (2011).

13 Jeong, S. E. *et al.* Genomic and metatranscriptomic analyses of *Weissella koreensis* reveal its metabolic and fermentative features during kimchi fermentation. *Food Microbiol* **76**, 1-10, doi:10.1016/j.fm.2018.04.003 (2018).

14 Jung, J. Y., Chun, B. H., Moon, J. Y., Yeo, S. H. & Jeon, C. O. Complete genome sequence of *Bacillus methylotrophicus* JJ-D34 isolated from deonjang, a Korean traditional fermented soybean paste. *J Biotechnol* **219**, 36-37, doi:10.1016/j.jbiotec.2015.12.030 (2016).

15 Lee, H. J., Chun, B. H., Jeon, H. H., Kim, Y. B. & Lee, S. H. Complete Genome Sequence of *Bacillus velezensis* YJ11-1-4, a Strain with Broad-Spectrum Antimicrobial Activity, Isolated from Traditional Korean Fermented Soybean Paste. *Genome Announc* **5**, doi:10.1128/genomeA.01352-17 (2017).

16 Shin, N. R. *et al.* Genome sequence of *Corynebacterium nuruki* S6-4^T^, isolated from alcohol fermentation starter. *J Bacteriol* **193**, 4257, doi:10.1128/JB.05354-11 (2011).

17 Kim, K. H. *et al.* *Acetobacter oryzifermentans* sp. nov., isolated from Korean traditional vinegar and reclassification of the type strains of *Acetobacter pasteurianus* subsp. *ascendens* (Henneberg 1898) and *Acetobacter pasteurianus* subsp. *paradoxus* (Frateur 1950) as *Acetobacter ascendens* sp. nov., comb. nov. *Syst Appl Microbiol* **41**, 324-332, doi:10.1016/j.syapm.2018.03.003 (2018).

18 Jia, B. *et al.* Complete Genome Sequences of Two Acetic Acid-Producing *Acetobacter pasteurianus* Strains (Subsp. *ascendens* LMG 1590^T^ and Subsp. paradoxus LMG 1591^T^). *Front Bioeng Biotechnol* **5**, 33, doi:10.3389/fbioe.2017.00033 (2017).

19 Jung, J., Chun, J. & Park, W. Genome sequence of extracellular-protease-producing *Alishewanella jeotgali* isolated from traditional Korean fermented seafood. *J Bacteriol* **194**, 2097, doi:10.1128/JB.00153-12 (2012).

20 Park, S. K., Roh, S. W., Whon, T. W. & Bae, J. W. Genome sequence of *Brachybacterium squillarum* M-6-3^T^, isolated from salt-fermented seafood. *J Bacteriol* **193**, 6416-6417, doi:10.1128/JB.06183-11 (2011).

21 Kim, J., Roh, S. W. & Bae, J. W. Draft genome sequence of *Dietzia alimentaria* 72^T^, belonging to the family Dietziaceae, isolated from a traditional Korean food. *J Bacteriol* **193**, 6791, doi:10.1128/JB.06229-11 (2011).

22 Jung, M. J., Roh, S. W., Kim, M. S., Whon, T. W. & Bae, J. W. Genome sequence of *Lentibacillus jeotgali* Grbi^T^, isolated from traditional Korean salt-fermented seafood. *J Bacteriol* **193**, 6414-6415, doi:10.1128/JB.06139-11 (2011).

23 Kim, J. *et al.* *Paracoccus jeotgali* sp. nov., isolated from Korean salted and fermented shrimp. *J Microbiol* **57**, 444-449, doi:10.1007/s12275-019-8704-8 (2019).

24 Lee, S. H., Jung, J. Y. & Jeon, C. O. Draft genome sequence of *Salimicrobium* sp. strain MJ3, isolated from Myulchi-Jeot, Korean fermented seafood. *J Bacteriol* **194**, 6695, doi:10.1128/JB.01808-12 (2012).

25 Sung, J. S., Chun, J., Choi, S. & Park, W. Genome sequence of the halotolerant *Staphylococcus* sp. strain OJ82, isolated from Korean traditional salt-fermented seafood. *J Bacteriol* **194**, 6353-6354, doi:10.1128/JB.01653-12 (2012).

26 Kim, K. H., Lee, S. H., Chun, B. H., Jeong, S. E. & Jeon, C. O. *Tetragenococcus halophilus* MJ4 as a starter culture for repressing biogenic amine (cadaverine) formation during saeu-jeot (salted shrimp) fermentation. *Food Microbiol* **82**, 465-473, doi:10.1016/j.fm.2019.02.017 (2019).

27 Cleenwerck, I., Vandemeulebroecke, K., Janssens, D. & Swings, J. Re-examination of the genus *Acetobacter*, with descriptions of *Acetobacter cerevisiae* sp. nov. and *Acetobacter malorum* sp. nov. *Int J Syst Evol Microbiol* **52**, 1551-1558, doi:10.1099/00207713-52-5-1551 (2002).

28 Lisdiyanti, P. *et al.* Systematic study of the genus *Acetobacter* with descriptions of *Acetobacter indonesiensis* sp. nov., *Acetobacter tropicalis* sp. nov., *Acetobacter orleanensis* (Henneberg 1906) comb. nov., *Acetobacter lovaniensis* (Frateur 1950) comb. nov., and *Acetobacter estunensis* (Carr 1958) comb. nov. *J Gen Appl Microbiol* **46**, 147-165, doi:10.2323/jgam.46.147 (2000).

29 Huang, C. H., Chang, M. T., Huang, L. & Chua, W. S. Molecular discrimination and identification of *Acetobacter* genus based on the partial heat shock protein 60 gene (hsp60) sequences. *J Sci Food Agric* **94**, 213-218, doi:10.1002/jsfa.6231 (2014).

30 Tohno, M. *et al.* Description of *Lactobacillus iwatensis* sp. nov., isolated from orchardgrass (Dactylis glomerata L.) silage, and *Lactobacillus backii* sp. nov. *Int J Syst Evol Microbiol* **63**, 3854-3860, doi:10.1099/ijs.0.051920-0 (2013).

31 Fraunhofer, M. E. *et al.* Characterization of beta-glucan formation by *Lactobacillus brevis* TMW 1.2112 isolated from slimy spoiled beer. *Int J Biol Macromol* **107**, 874-881, doi:10.1016/j.ijbiomac.2017.09.063 (2018).

32 Long, G. Y. & Gu, C. T. *Lactobacillus jixianensis* sp. nov., *Lactobacillus baoqingensis* sp. nov., *Lactobacillus jiayinensis* sp. nov., *Lactobacillus zhaoyuanensis* sp. nov., *Lactobacillus lindianensis* sp. nov., *Lactobacillus huananensis* sp. nov., *Lactobacillus tangyuanensis* sp. nov., *Lactobacillus fuyuanensis* sp. nov., *Lactobacillus tongjiangensis* sp. nov., *Lactobacillus fujinensis* sp. nov. and *Lactobacillus mulengensis* sp. nov., isolated from Chinese traditional pickle. *Int J Syst Evol Microbiol* **69**, 2340-2353, doi:10.1099/ijsem.0.003474 (2019).

33 Geissler, A. J., Behr, J., von Kamp, K. & Vogel, R. F. Metabolic strategies of beer spoilage lactic acid bacteria in beer. *Int J Food Microbiol* **216**, 60-68, doi:10.1016/j.ijfoodmicro.2015.08.016 (2016).

34 Bergsveinson, J., Thomson, E., Jacoby, D., Coady, Y. & Ziola, B. Genome Sequence of *Megasphaera cerevisiae* NSB1, a Bacterium Isolated from a Canning Line and Able To Grow in Beer with High Alcohol Content. *Genome Announc* **5**, doi:10.1128/genomeA.01686-16 (2017).

35 Kramer, T. *et al.* Comparative genetic and physiological characterisation of *Pectinatus* species reveals shared tolerance to beer-associated stressors but halotolerance specific to pickle-associated strains. *Food Microbiol* **90**, 103462, doi:10.1016/j.fm.2020.103462 (2020).

36 Kern, C. C., Usbeck, J. C., Vogel, R. F. & Behr, J. Optimization of Matrix-Assisted-Laser-Desorption-Ionization-Time-Of-Flight Mass Spectrometry for the identification of bacterial contaminants in beverages. *J Microbiol Methods* **93**, 185-191, doi:10.1016/j.mimet.2013.03.012 (2013).

37 Kajala, I. *et al.* *Lactobacillus backii* and *Pediococcus damnosus* isolated from 170-year-old beer recovered from a shipwreck lack the metabolic activities required to grow in modern lager beer. *FEMS Microbiol Ecol* **94**, doi:10.1093/femsec/fix152 (2018).

38 Storari, M., Wuthrich, D., Bruggmann, R., Berthoud, H. & Arias-Roth, E. Draft Genome Sequences of *Clostridium tyrobutyricum* Strains FAM22552 and FAM22553, Isolated from Swiss Semihard Red-Smear Cheese. *Genome Announc* **3**, doi:10.1128/genomeA.00078-15 (2015).

39 Collins, M. D., Falsen, E., Akervall, E., Sjoden, B. & Alvarez, A. *Corynebacterium kroppenstedtii* sp. nov., a novel corynebacterium that does not contain mycolic acids. *Int J Syst Bacteriol* **48**, 1449-1454, doi:10.1099/00207713-48-4-1449 (1998).

40 Martino, G. P., Quintana, I. M., Espariz, M., Blancato, V. S. & Magni, C. Aroma compounds generation in citrate metabolism of *Enterococcus faecium*: Genetic characterization of type I citrate gene cluster. *Int J Food Microbiol* **218**, 27-37, doi:10.1016/j.ijfoodmicro.2015.11.004 (2016).

41 Bhakdi, S., Klonisch, T., Nuber, P. & Fischer, W. Stimulation of monokine production by lipoteichoic acids. *Infect Immun* **59**, 4614-4620, doi:10.1128/IAI.59.12.4614-4620.1991 (1991).

42 Bonham, K. S., Wolfe, B. E. & Dutton, R. J. Extensive horizontal gene transfer in cheese-associated bacteria. *Elife* **6**, doi:10.7554/eLife.22144 (2017).

43 Oguntoyinbo, F. A. *et al.* *Halomonas nigrificans* sp. nov., isolated from cheese. *Int J Syst Evol Microbiol* **68**, 371-376, doi:10.1099/ijsem.0.002515 (2018).

44 Zhuravleva, D. E. *et al.* Complete Genome Sequence of *Lactobacillus hilgardii* LMG 7934, Carrying the Gene Encoding for the Novel PII-Like Protein PotN. *Curr Microbiol* **77**, 3538-3545, doi:10.1007/s00284-020-02161-6 (2020).

45 Hynonen, U. *et al.* Functional characterization of probiotic surface layer protein-carrying *Lactobacillus amylovorus* strains. *BMC Microbiol* **14**, 199, doi:10.1186/1471-2180-14-199 (2014).

46 Pepper, S. J. & Britz, M. L. An Acid Up-Regulated Surface Protein of *Lactobacillus paracasei* Strain GCRL 46 is Phylogenetically Related to the Secreted Glucan- (GpbB) and Immunoglobulin-Binding (SibA) Protein of Pathogenic Streptococci. *Int J Mol Sci* **20**, doi:10.3390/ijms20071610 (2019).

47 Somerville, V. *et al.* Long-read based de novo assembly of low-complexity metagenome samples results in finished genomes and reveals insights into strain diversity and an active phage system. *BMC Microbiol* **19**, 143, doi:10.1186/s12866-019-1500-0 (2019).

48 Ennahar, S. *et al.* Production of pediocin AcH by *Lactobacillus plantarum* WHE 92 isolated from cheese. *Appl Environ Microbiol* **62**, 4381-4387, doi:10.1128/AEM.62.12.4381-4387.1996 (1996).

49 Tschoeke, D. A. *et al.* Exploring the Genome of Cheese Starter Lactic Acid Bacterium *Lactococcus lactis* subsp. *lactis* CECT 4433. *Genome Announc* **2**, doi:10.1128/genomeA.01142-14 (2014).

50 Frantzen, C. A. *et al.* Genomic Characterization of Dairy Associated Leuconostoc Species and Diversity of Leuconostocs in Undefined Mixed Mesophilic Starter Cultures. *Front Microbiol* **8**, 132, doi:10.3389/fmicb.2017.00132 (2017).

51 Makela, P., Schillinger, U., Korkeala, H. & Holzapfel, W. H. Classification of ropy slime-producing lactic acid bacteria based on DNA-DNA homology, and identification of *Lactobacillus sake* and *Leuconostoc amelibiosum* as dominant spoilage organisms in meat products. *Int J Food Microbiol* **16**, 167-172, doi:10.1016/0168-1605(92)90011-q (1992).

52 Guo, X. *et al.* Detection and Genomic Characterization of a *Morganella morganii* Isolate From China That Produces NDM-5. *Front Microbiol* **10**, 1156, doi:10.3389/fmicb.2019.01156 (2019).

53 Li, J. *et al.* *Mycetocola zhujimingii* sp. nov., isolated from faeces of Tibetan antelopes (Pantholops hodgsonii). *Int J Syst Evol Microbiol* **69**, 1117-1122, doi:10.1099/ijsem.0.003280 (2019).

54 Quintieri, L. *et al.* Biofilm and Pathogenesis-Related Proteins in the Foodborne *P. fluorescens* ITEM 17298 With Distinctive Phenotypes During Cold Storage. *Front Microbiol* **11**, 991, doi:10.3389/fmicb.2020.00991 (2020).

55 Quintieri, L., Caputo, L., De Angelis, M. & Fanelli, F. Genomic Analysis of Three Cheese-Borne *Pseudomonas lactis* with Biofilm and Spoilage-Associated Behavior. *Microorganisms* **8**, doi:10.3390/microorganisms8081208 (2020).

56 Bertuzzi, A. S. *et al.* Genome Sequence of *Staphylococcus saprophyticus* DPC5671, a Strain Isolated from Cheddar Cheese. *Genome Announc* **5**, doi:10.1128/genomeA.00193-17 (2017).

57 Wels, M. *et al.* Draft Genome Sequence of *Streptococcus thermophilus* C106, a Dairy Isolate from an Artisanal Cheese Produced in the Countryside of Ireland. *Genome Announc* **3**, doi:10.1128/genomeA.01377-15 (2015).

58 Toh, H. *et al.* Complete genome sequence of *Lactobacillus acetotolerans* RIB 9124 (NBRC 13120) isolated from putrefied (hiochi) Japanese sake. *J Biotechnol* **214**, 214-215, doi:10.1016/j.jbiotec.2015.09.006 (2015).

59 Gong, L. *et al.* A New Isolate of *Pediococcus pentosaceus* (SL001) With Antibacterial Activity Against Fish Pathogens and Potency in Facilitating the Immunity and Growth Performance of Grass Carps. *Front Microbiol* **10**, 1384, doi:10.3389/fmicb.2019.01384 (2019).

60 He, Q. *et al.* Comparative genomic analysis of *Enterococcus faecalis*: insights into their environmental adaptations. *BMC Genomics* **19**, 527, doi:10.1186/s12864-018-4887-3 (2018).

61 Iartchouk, O., Kozyavkin, S., Karamychev, V. & Slesarev, A. Complete Genome Sequence of *Lactobacillus acidophilus* FSI4, Isolated from Yogurt. *Genome Announc* **3**, doi:10.1128/genomeA.00166-15 (2015).

62 Laino, J. E., Hebert, E. M., Savoy de Giori, G. & LeBlanc, J. G. Draft Genome Sequence of *Lactobacillus delbrueckii* subsp. *bulgaricus* CRL871, a Folate-Producing Strain Isolated from a Northwestern Argentinian Yogurt. *Genome Announc* **3**, doi:10.1128/genomeA.00693-15 (2015).

63 Bai, Y. *et al.* Complete genome sequence of *Streptococcus thermophilus* MN-BM-A01, a strain with high exopolysaccharides production. *J Biotechnol* **224**, 45-46, doi:10.1016/j.jbiotec.2016.03.003 (2016).

64 Lee, H. W. *et al.* Draft genome sequence of the extremely halophilic archaeon *Haladaptatus cibarius* type strain D43^T^ isolated from fermented seafood. *Stand Genomic Sci* **10**, 53, doi:10.1186/s40793-015-0051-8 (2015).

65 Roh, S. W. *et al.* Complete genome sequence of *Halalkalicoccus jeotgali* B3^T^, an extremely halophilic archaeon. *J Bacteriol* **192**, 4528-4529, doi:10.1128/JB.00663-10 (2010).

66 Song, H. S. *et al.* Draft genome sequence of *Halapricum salinum* CBA1105^T^, an extremely halophilic archaeon isolated from solar salt. *Mar Genomics* **18**, 133-134, doi:10.1016/j.margen.2014.09.006 (2014).

67 Yun, J. H. *et al.* Complete genome sequence of *Haloarcula* sp. CBA1115 isolated from non-purified solar salts. *Mar Genomics* **23**, 19-21, doi:10.1016/j.margen.2015.03.012 (2015).

68 Lim, S. K. *et al.* Genomic Analysis of the Extremely Halophilic Archaeon *Halobacterium noricense* CBA1132 Isolated from Solar Salt That Is an Essential Material for Fermented Foods. *J Microbiol Biotechnol* **26**, 1375-1382, doi:10.4014/jmb.1603.03010 (2016).

69 Lee, M. H. *et al.* Draft genome sequence of the agarolytic haloarchaeon *Halobellus rufus* type strain CBA1103. *FEMS Microbiol Lett* **362**, 1-3, doi:10.1093/femsle/fnu005 (2015).

70 Yim, K. J. *et al.* Draft genome sequence of the extremely halophilic archaeon *Halococcus sediminicola* CBA1101^T^ isolated from a marine sediment sample. *Mar Genomics* **18**, 145-146, doi:10.1016/j.margen.2014.10.003 (2014).

71 Lee, M. H. *et al.* Draft genome sequence of *Halolamina rubra* CSA1107^T^, an agarolytic haloarchaeon isolated from solar salt. *Mar Genomics* **18**, 127-128, doi:10.1016/j.margen.2014.09.003 (2014).

72 Lee, H. W. *et al.* Draft genome sequence of *Halorubrum halophilum* B8^T^, an extremely halophilic archaeon isolated from salt-fermented seafood. *Mar Genomics* **18**, 117-118, doi:10.1016/j.margen.2014.08.005 (2014).

73 Cha, I. T. *et al.* Genome sequence of the haloarchaeon *Haloterrigena jeotgali* type strain A29^T^ isolated from salt-fermented food. *Stand Genomic Sci* **10**, 49, doi:10.1186/s40793-015-0047-4 (2015).

74 Kim, Y. B. *et al.* Novel haloarchaeon *Natrinema thermophila* having the highest growth temperature among haloarchaea with a large genome size. *Sci Rep* **8**, 7777, doi:10.1038/s41598-018-25887-7 (2018).

75 Kim, J. Y. *et al.* Community structures and genomic features of undesirable white colony-forming yeasts on fermented vegetables. *J Microbiol* **57**, 30-37, doi:10.1007/s12275-019-8487-y (2019).

76 Gallone, B. *et al.* Domestication and Divergence of *Saccharomyces cerevisiae* Beer Yeasts. *Cell* **166**, 1397-1410 e1316, doi:10.1016/j.cell.2016.08.020 (2016).

77 Salazar, A. N. *et al.* Chromosome level assembly and comparative genome analysis confirm lager-brewing yeasts originated from a single hybridization. *BMC Genomics* **20**, 916, doi:10.1186/s12864-019-6263-3 (2019).

78 Nguyen, H. V. & Gaillardin, C. Evolutionary relationships between the former species *Saccharomyces uvarum* and the hybrids *Saccharomyces bayanus* and *Saccharomyces pastorianus*; reinstatement of *Saccharomyces uvarum* (Beijerinck) as a distinct species. *FEMS Yeast Res* **5**, 471-483, doi:10.1016/j.femsyr.2004.12.004. (2005).

79 Rainieri, S., Kodama, Y., Nakao, Y., Pulvirenti, A. & Giudici, P. The inheritance of mtDNA in lager brewing strains. *FEMS Yeast Res* **8**, 586-596, doi:10.1111/j.1567-1364.2008.00363.x (2008).

80 Hewitt, S. K., Donaldson, I. J., Lovell, S. C. & Delneri, D. Sequencing and characterisation of rearrangements in three *S. pastorianus* strains reveals the presence of chimeric genes and gives evidence of breakpoint reuse. *PLoS One* **9**, e92203, doi:10.1371/journal.pone.0092203 (2014).

81 Tafer, H., Sterflinger, K. & Lopandic, K. Draft Genome Sequence of the Interspecies Hybrid *Saccharomyces pastorianus* Strain HA2560, Isolated from a Municipal Wastewater Treatment Plant. *Genome Announc* **6**, doi:10.1128/genomeA.00341-18 (2018).

82 Gibson, B. & Liti, G. *Saccharomyces pastorianus*: genomic insights inspiring innovation for industry. *Yeast* **32**, 17-27, doi:10.1002/yea.3033 (2015).

83 Bing, J., Han, P. J., Liu, W. Q., Wang, Q. M. & Bai, F. Y. Evidence for a Far East Asian origin of lager beer yeast. *Curr Biol* **24**, R380-381, doi:10.1016/j.cub.2014.04.031 (2014).

84 Jung, J. Y. *et al.* Effects of *Leuconostoc mesenteroides* starter cultures on microbial communities and metabolites during kimchi fermentation. *Int J Food Microbiol* **153**, 378-387, doi:10.1016/j.ijfoodmicro.2011.11.030 (2012).

85 Jeong, S. H., Jung, J. Y., Lee, S. H., Jin, H. M. & Jeon, C. O. Microbial succession and metabolite changes during fermentation of dongchimi, traditional Korean watery kimchi. *Int J Food Microbiol* **164**, 46-53, doi:10.1016/j.ijfoodmicro.2013.03.016 (2013).

86 Jeong, S. H. *et al.* Effects of red pepper powder on microbial communities and metabolites during kimchi fermentation. *Int J Food Microbiol* **160**, 252-259, doi:10.1016/j.ijfoodmicro.2012.10.015 (2013).

87 Jeong, S. H., Lee, S. H., Jung, J. Y., Choi, E. J. & Jeon, C. O. Microbial succession and metabolite changes during long-term storage of Kimchi. *J Food Sci* **78**, M763-769, doi:10.1111/1750-3841.12095 (2013).

88 Lee, S. H., Jung, J. Y. & Jeon, C. O. Source Tracking and Succession of Kimchi Lactic Acid Bacteria during Fermentation. *J Food Sci* **80**, M1871-1877, doi:10.1111/1750-3841.12948 (2015).

89 Jung, M. Y. *et al.* Role of jeotgal, a Korean traditional fermented fish sauce, in microbial dynamics and metabolite profiles during kimchi fermentation. *Food Chem* **265**, 135-143, doi:10.1016/j.foodchem.2018.05.093 (2018).

90 Jung, J. Y. *et al.* Metatranscriptomic analysis of lactic acid bacterial gene expression during kimchi fermentation. *Int J Food Microbiol* **163**, 171-179, doi:10.1016/j.ijfoodmicro.2013.02.022 (2013).

91 Song, H. S. *et al.* Microbial niches in raw ingredients determine microbial community assembly during kimchi fermentation. *Food Chem* **318**, 126481, doi:10.1016/j.foodchem.2020.126481 (2020).

92 Jung, J. Y., Lee, S. H. & Jeon, C. O. Microbial community dynamics during fermentation of doenjang-meju, traditional Korean fermented soybean. *Int J Food Microbiol* **185**, 112-120, doi:10.1016/j.ijfoodmicro.2014.06.003 (2014).

93 Jung, W. Y., Jung, J. Y., Lee, H. J. & Jeon, C. O. Functional Characterization of Bacterial Communities Responsible for Fermentation of Doenjang: A Traditional Korean Fermented Soybean Paste. *Front Microbiol* **7**, 827, doi:10.3389/fmicb.2016.00827 (2016).

94 Chun, B. H., Kim, K. H., Jeong, S. E. & Jeon, C. O. The effect of salt concentrations on the fermentation of doenjang, a traditional Korean fermented soybean paste. *Food Microbiol* **86**, 103329, doi:10.1016/j.fm.2019.103329 (2020).

95 Jung, J. Y., Lee, S. H., Lee, H. J. & Jeon, C. O. Microbial succession and metabolite changes during fermentation of saeu-jeot: traditional Korean salted seafood. *Food Microbiol* **34**, 360-368, doi:10.1016/j.fm.2013.01.009 (2013).

96 Lee, S. H., Jung, J. Y. & Jeon, C. O. Effects of temperature on microbial succession and metabolite change during saeu-jeot fermentation. *Food Microbiol* **38**, 16-25, doi:10.1016/j.fm.2013.08.004 (2014).

97 Lee, S. H., Jung, J. Y. & Jeon, C. O. Microbial successions and metabolite changes during fermentation of salted shrimp (saeu-jeot) with different salt concentrations. *PLoS One* **9**, e90115, doi:10.1371/journal.pone.0090115 (2014).

98 Lee, S. H., Jung, J. Y. & Jeon, C. O. Bacterial community dynamics and metabolite changes in myeolchi-aekjeot, a Korean traditional fermented fish sauce, during fermentation. *Int J Food Microbiol* **203**, 15-22, doi:10.1016/j.ijfoodmicro.2015.02.031 (2015).

99 Jung, J. Y., Lee, H. J., Chun, B. H. & Jeon, C. O. Effects of Temperature on Bacterial Communities and Metabolites during Fermentation of Myeolchi-Aekjeot, a Traditional Korean Fermented Anchovy Sauce. *PLoS One* **11**, e0151351, doi:10.1371/journal.pone.0151351 (2016).

100 Jung, M. J. *et al.* Viral community predicts the geographical origin of fermented vegetable foods more precisely than bacterial community. *Food Microbiol* **76**, 319-327, doi:10.1016/j.fm.2018.06.010 (2018).

101 Jung, J. Y. *et al.* Metagenomic analysis of kimchi, a traditional Korean fermented food. *Appl Environ Microbiol* **77**, 2264-2274, doi:10.1128/AEM.02157-10 (2011).
